# One-dimensional dielectric grating structure for plasmonic coupling and routing

**DOI:** 10.1515/nanoph-2025-0506

**Published:** 2025-12-05

**Authors:** Lam Yen Thi Nguyen, Yu-Cheng Lin, Tzu-Yu Chiu, Shao-Jin Liao, Chia-Chen Hsu, Jiunn-Yuan Lin, Hung-Chih Kan

**Affiliations:** Department of Physics, 34915 National Chung Cheng University, 168, Sec. 1, University Road, Min-Hsiung, Chiayi, 621, Taiwan

**Keywords:** surface plasmon polaritons, plasmonic 1-D grating structures, plasmonic coupling, plasmonic routing

## Abstract

We propose and demonstrate one-dimensional (1-D) TiO_2_ dielectric grating structures that couple 793-nm wavelength light and two-dimensional (2-D) surface plasmon polaritons (SPPs) into guided 1-D SPPs supported by dielectric-loaded plasmonic waveguides. The 1-D grating structure consists of a central TiO_2_ stripe with a periodic array of TiO_2_ teeth attached to the stripe. Finite-difference time-domain (FDTD) simulations reveal that the electromagnetic boundary conditions created by the teeth bend the electric field and induce charge oscillations under the grating, enabling excitation of SPPs. The same mechanism supports the routing of 2-D SPP. In the simulation the symmetric gratings achieve a maximum coupling efficiency of 19.1 % at an optimized grating period of Λ = 600 nm, and 1.7 % for asymmetric gratings. Both types exhibit strong polarization selectivity: symmetric gratings couple only under TM excitation, whereas asymmetric gratings respond under TE excitation. Experimental confirms these behaviors, yielding a coupling efficiency of ∼13 % for optimized symmetric gratings. The structures also function as SPP routers. Asymmetric gratings route incoming 2-D SPPs into 1-D TiO_2_ waveguides with a simulated routing efficiency of 5.7 %, compared to 4.0 % for symmetric designs. The devices offer a ∼14 nm bandwidth around 793 nm and a small footprint of 18.7 μm^2^, resulting in a figure of merit (efficiency/area) of 0.71 % μm^−2^, the highest among reported devices designed to couple free-space light directly into 1-D SPP waveguides. These results demonstrate that 1-D TiO_2_ gratings offer a compact and multifunctional platform for efficient coupling and routing of SPPs in integrated plasmonic circuits.

## 1 Introduction

Surface plasmon polaritons (SPPs) are electromagnetic waves that propagate at the interface between metal and dielectric materials [1]. Their *E*-field intensity is greatly enhanced and confined at the interface. This has invoked enormous research interest in the control of the SPP propagation at subwavelength scale for guiding and manipulation of light for promising applications, such as information processing in integrated plasmonic circuit [2], [3], optoelectronic devices, waveguides [4], [5], modulator [6], directional couplers [7], [8], switches [9], [10], and generalized sensors [11], [12], [13]. As a first stage of the development of plasmonic applications, there have been numerous investigations focused on the excitation of SPPs using various designs, including gratings [14], [15], [16], [17], [18], [19], [20], [21], [22], [23], [24], ridges [25], slits, and single defects [26], [27], [28], [29], [30], [31], [32] on metallic film. In general, these metallic structures have been reported to couple the incident light to two-dimensional (2-D) SPPs with sufficient efficiencies. The subsequent stages then involve delivering the excited SPP to devices to perform designated functions. This leads to the development of various plasmonic waveguides, such as nano-groove on the metal film [33], [34], [35], [36], and a dielectric-loaded SPP waveguide [37], [38], [39], [40]. However, due to the fact that most of the proposed waveguide structures are one-dimensional (1-D), it is important to develop efficient mechanisms for exciting SPPs that is readily coupled to these 1-D plasmonic waveguides [37], [39]. Furthermore, for the goal of the continual miniaturization of the device structures, it is natural to develop 1-D structures with smallest possible foot print to effectively couple the incident light to the SPP waveguides with sufficient efficiency and small foot-print. In addition, the development of multifunctional structures to control SPP propagation also represents a significant progression for the next generation of plasmonic devices [41], [42], [43].

In this paper, we propose and demonstrate the 1-D TiO_2_ grating structures for coupling of 793-nm-wavelength incident light as well as its corresponding 2-D SPPs to SPPs propagating along a dielectric loaded 1-D plasmonic waveguide. The 1-D TiO_2_ grating consists of arrays of rectangular teeth attached to both sides of the central stripe in either a symmetric or an asymmetric manner. We carried out numerical simulations with finite difference time domain (FDTD) method to investigate and optimize the SPP coupling with the 1-D grating structures. Based on our numerical results, the TiO_2_ teeth set the boundary condition for the incident light to bend the E-field toward/away from the SiO_2_/Ag interface, which induce charge oscillation with opposite phase in an alternating fashion. This results in the excitation of SPP under the grating structure. From the simulation, we obtain that the efficiency of SPP coupling with the optimal symmetric grating structure is ∼19 %, and 1.7 % for the asymmetric structure. We also fabricated the 1-D grating and the dielectric loaded SPP waveguide structures and characterized the SPP coupling with up-conversion fluorescence microscopy. We obtained qualitatively agreement between the simulation and the experiment. Furthermore, we demonstrate the versatility of the 1-D grating structures, in particular its ability to function as an SPP router that couples the 2-D SPP wave to the dielectric loaded 1-D plasmonic waveguide. We conducted both numerical and experimental investigations to demonstrate the SPP routing of the 1-D grating structures. The efficiency of the SPP routing was shown to be higher for the asymmetric grating structure (5.7 %) compared to that of the symmetric grating structure(4.0 %). For the performance of light-SPP coupling, both the coupling efficiency and the miniaturization of the device play important roles in the advance of plasmonics and nanotechnology. To quantify the performance, we define the figure of merit (FOM) to be the ratio between the efficiency and the lateral size of the device. In comparison with the FOMs of the device reported in the literature, our optimal 1-D grating achieves the highest FOM for the case of coupling incident light directly to SPP propagating along a 1-D waveguide.

## 2 Simulation and experimental method

### 2.1 Sample design and fabrication

**Figure 1:**
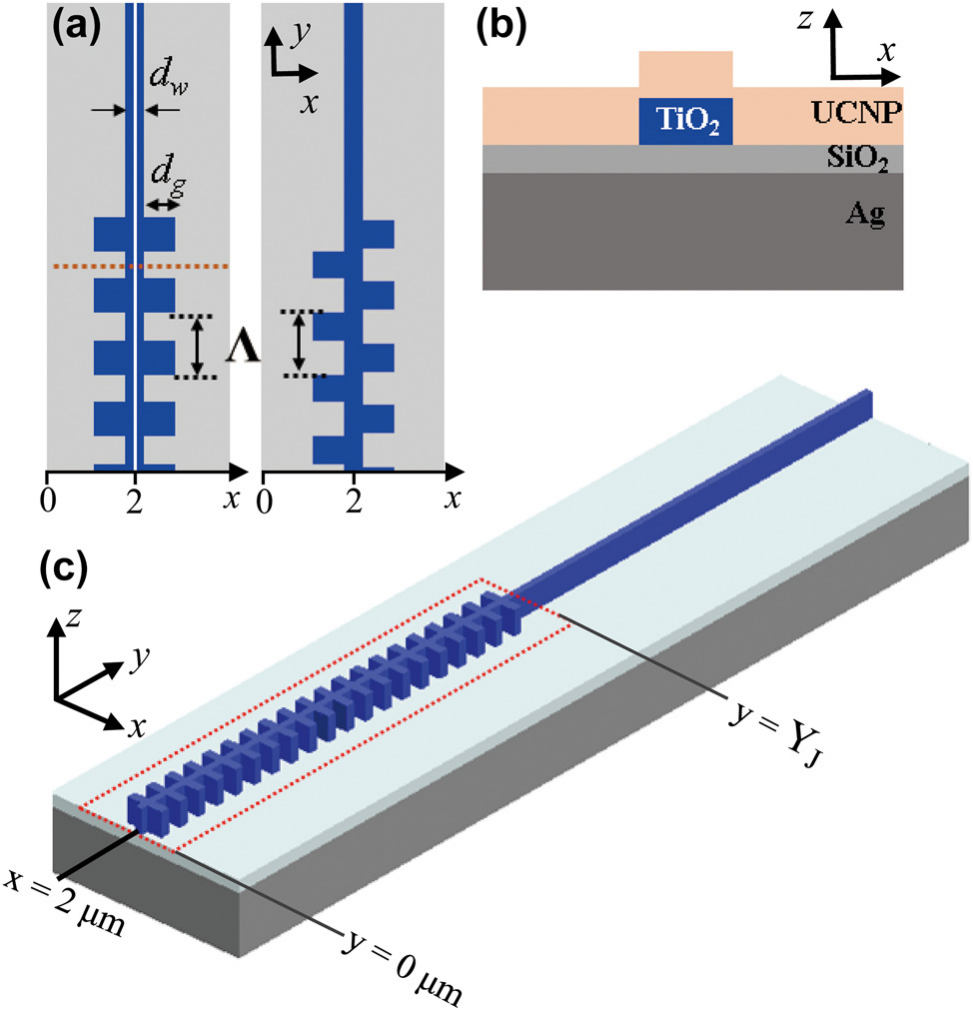
Schematics of 1-D TiO_2_ grating structures for SPP coupling. (a) The left and the right panels show the top view of symmetric and asymmetric grating structures, respectively. (b) The cross-sectional view of the sample structure across the horizontal dashed line in (a). (c) The 3D perspective view of the symmetric TiO_2_ grating structure. For clarity, the UCNP layer is not shown in (a) and (c).

Figure 1 shows the schematics of the design of the 1-D TiO_2_ grating structures and the dielectric loaded SPP waveguide. The top view of the 1-D TiO_2_ grating structures is shown in Figure 1(a). The TiO_2_ grating structures consist of the symmetric or asymmetric arrangement of arrays of rectangular grating teeth, where the grating tooth width is denoted by ***d***_***g***_ and the period **Λ**. These teeth are attached to both sides of the central TiO_2_ stripe, with the width of ***d***_***w***_. At the upper end of the grating structures, the extending central stripes were designated as the dielectric loaded SPP waveguide, which we refer to as “TiO_2_ waveguide” below. Figure 1(b) shows the cross-sectional view of the 1-D grating structure. The 1-D TiO_2_ grating structures are placed on top of the SiO_2_/Ag interface. The SiO_2_ layer has a thickness of 20 nm, and the thickness of the TiO_2_ structures is 40 nm. For ease of fabrication, the 1-D grating and the TiO_2_ waveguide structure are designed to be fabricated in a single EBL step, i.e. both consist of TiO_2_ structure with the same thickness. In the optimization process, we aimed to achieve the maximum SPP signal strength at the junction of the 1-D grating and the waveguide. For implementing the up-conversion fluorescence microscopy imaging, a monolayer of NaYF_4_:Yb^3+^, Er^3+^@ NaYF_4_:Yb^3+^, Nd^3+^@ NaYF_4_ core-double-shell up-conversion nanoparticles (UCNPs) were coated on the surface of the sample. The nominal thickness of the UCNP layer is ∼50 nm. Figure 1(c) illustrates a three-dimensional (3-D) perspective view of the symmetric TiO_2_ grating structure on the SiO_2_/Ag substrate. In our investigation, the length of the grating structure is fixed to 40**Λ**, i.e. from ***y*** = 0 to ***y*** = *Y*_***J***_. For SPP coupling, the incident light selectively illuminates the grating structures only, which is indicated by the dashed rectangle.

A brief description of the sample fabrication procedure follows. First, we deposited 100 nm-thick Ag film on top of a clean ITO-glass substrate with a thermal evaporator. Next, we deposited a 20 nm-thick layer of SiO_2_ on top of the Ag film with an electron beam evaporator. The fabrication of symmetric and asymmetric grating structures was achieved via electron beam lithography (EBL). Initially, a poly(methyl methacrylate) (PMMA) film was spin-coated onto the SiO_2_/Ag/ITO-glass sample. Subsequently, the grating structures were patterned onto the PMMA film using EBL. We used the same electron beam evaporator to coat a layer of 40-nm-thick TiO_2_ film on top of the sample. After the lift-off process, the PMMA coating was removed, leaving the 1-D TiO_2_ grating structures on the sample. Finally, we spin coated a layer of UCNP on top of the sample. Fig. S1(a) shows a cross-sectional view of the device we fabricated on the ITO/glass substrate, and Fig. S2(b) and S2(c) shows the top view of the device after the spin-coating of the UCNPs. It is clear that the UCNPs uniformly covered the devices we fabricated.

### 2.2 Synthesis of NaYF_4_:Yb^3+^, Er^3+^@NaYF_4_:Yb^3+^, Nd^3+^@NaYF_4_ core@shell@shell UNCPs

NaYF_4_:Yb^3+^, Er^3+^@NaYF_4_:Yb^3+^, and Nd^3+^@NaYF_4_ core-shell-shell hexagonal-phase (β-phase) monodispersed nanocrystals were synthesized with a thermal decomposition method [44], [45], [46]. The UCNPs have an average diameter of ∼50 nm. Detailed description of the synthesis methods was reported previously [47], [48].

### 2.3 Up-conversion fluorescence microscopy

The configuration of our up-conversion fluorescence microscopy system is illustrated in Fig. S2. The system comprises the illumination system and the imaging system [49]. We utilized a collimated laser beam with a wavelength of 793 nm as the excitation light source. The selective illumination of the TiO_2_ grating structure on the sample was achieved with the spatial light modulator (SLM). Fluorescent signal of 549 nm wavelength emitted from the UCNPs was collected with a CCD camera to obtain images of the sample. Due to the shorter wavelength of the fluorescent signal compared to that of the SPP propagating on the sample, this enables the direct visualization of the SPP coupling with supreme resolution [47], [49].

### 2.4 Numerical simulation

In order to assist the design of the 1-D TiO_2_ grating structure, we performed numerical calculations to simulate the experiment with FDTD method by using commercially available software package (Lumerical Inc.). We constructed a 3-D model for the simulation based on the schematics shown in Figure 1. Refractive indices for Ag, SiO_2_, and TiO_2_ were acquired from the software’s own materials database, with the refractive index of the UCNP set at 1.57 [50]. In the simulation, the incident light was collimated and defined to illuminate only the 1-D grating structure area at normal incidence. We analyzed the simulated SPP coupling with the lateral *E*-field intensity *|E|* ^2^ distributions to optimize the performance of the 1-D TiO_2_ grating structures. The amplitude of the incident light E-field is set to unity. Therefore, the intensity plot of the *|E|* ^2^ is equivalent to the enhancement factor of the E-field. Due to limited computing resource and the intention to make the structure interface boundary to coincide with the grid mesh, we opt to use the grid size of 30 nm in the *x*- and *y*-direction, and 10 nm in the *z*-direction. Varying the grid size would leads small variation of the numerical value of the simulation but has minimal effect on the general trend of the data.

## 3 Results

**Figure 2:**
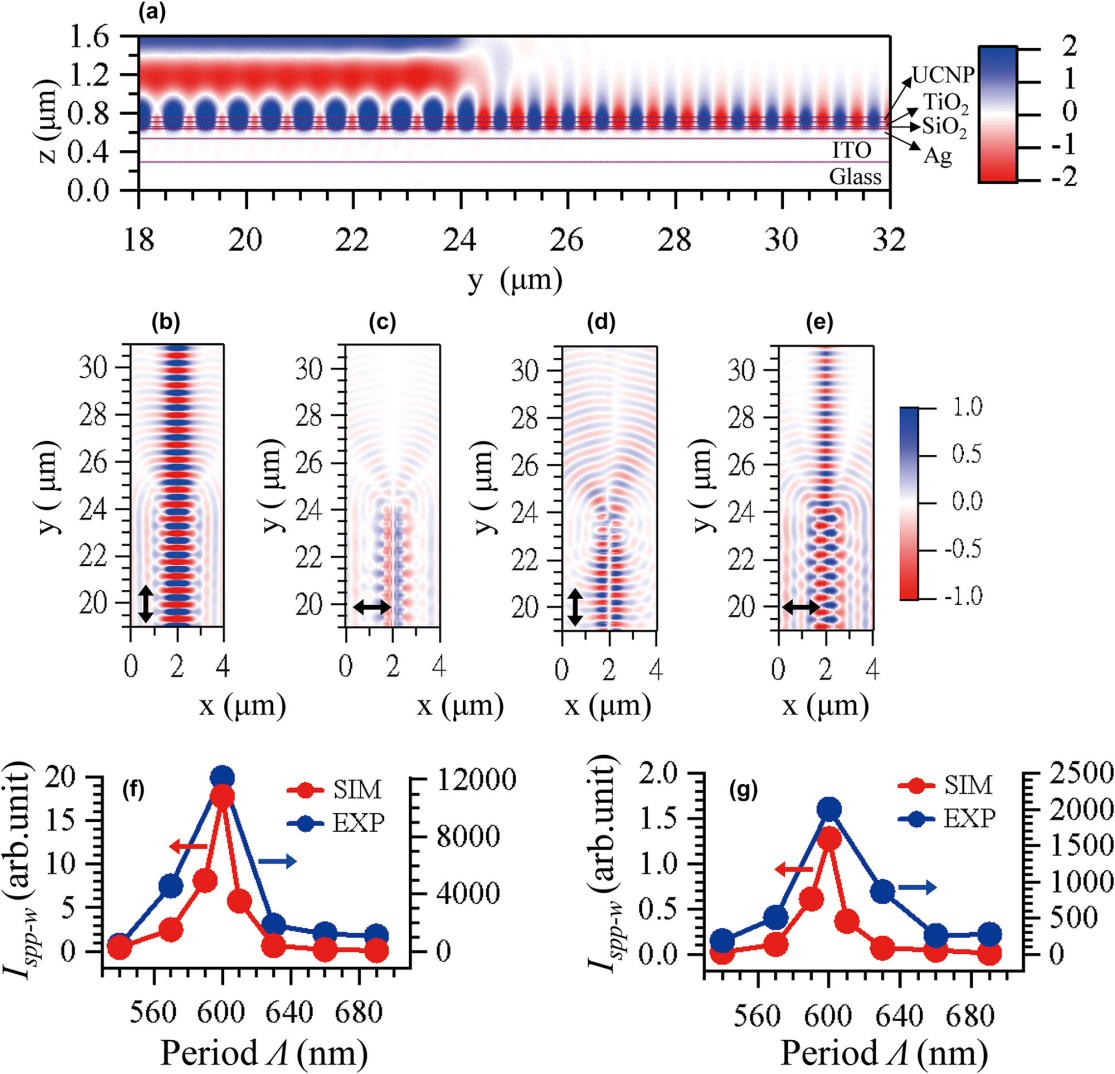
The SPP coupling of 1-D grating structures. (a) A snap shot of the simulated *E*_**y**_-component of the *E*-field distribution in the central *y-z* plane across the vertical dashed line in Figure 1(a) of the symmetric grating. The incident light of 793 nm wavelength illuminates the 1-D grating structure on the left. (b) and (c) The snap shot of the simulated *E*_**z**_-component of the *E*-field distribution at the SiO_2_/Ag interface under the symmetric 1-D grating, and (d), (e) the *E*_**z**_-component of the *E*-field distribution at the SiO_2_/Ag interface under the asymmetric 1-D grating. The polarization of the incident light is parallel to *y*-direction in (a), (b) and (d), and to the *x*-direction in (c) and (e). For the results in (a)–(e), the grating period **Λ** is 600 nm, ***d***_***g***_ = 300 nm, and ***d***_***w***_ = 180 nm. In (a), the period of the *E*_**z**_ distribution at the TiO_2_ waveguide region is 632 nm, which corresponds to the real part of the propagation constant of *β*_*R*_ = 9.94 μm^−1^. (g) and (f) The simulated (red circles) and experimental (blue circles) SPP intensity at the TiO_2_ waveguide as a function of the grating period **Λ** for the symmetric and asymmetric structures, respectively.

Figure 2 shows the results of the FDTD simulation for the SPP coupling of the 1-D grating structures. Figure 2(a) and (b) show the typical SPP coupling of the symmetric 1-D grating structure with the incident light polarization in the *y*-direction, i.e. transverse magnetic (TM) mode. Figure 2(a) shows the cross-sectional view of the *E*_**y**_-component of the *E*-field in the central y-z plane normal to the sample surface. For ease of observation, we show an enlarged view of the *E*-field distribution at the region near the junction (*y* = *Y*_***J***_) of the 1-D grating and the TiO_2_ waveguide. On the left-hand side of Figure 2(a) (*y* ≤ 24 μm) the incident light reaches the symmetric 1-D grating structure from the top at normal incidence. The incident light resonates with the TiO_2_ grating structure and results in periodic distribution of *E*_**y**_ at the sample surface, which further becomes propagating waves in the *y*-direction into the TiO_2_ waveguide (*y* ≥ 24 μm). Fig. S3 shows the distribution of the time-averaged Poynting vector on the same plane. It clearly indicates that the grating structure transfers the downward flux of the incident light energy to the flux along the direction of SPP propagation. In Figure 2(a) the presence of the longitudinal *E*-field component (*E*_**y**_) signatures the propagation of SPP at the SiO_2_/Ag interface under the TiO_2_ waveguide. Figure 2(b) shows the corresponding *E*_**z**_-component distribution at the SiO_2_/Ag interface. It is clear that the grating couples the incident light into SPPs, which propagate upward (*y*-direction) along the TiO_2_ waveguide. We then explore the incident light polarization dependence of the SPP coupling. Figure 2(c) shows that for the transverse electric mode (TE) incidence, the *E*_**z**_ component of SPP at the SiO_2_/Ag interface become minimal at the TiO_2_ waveguide region. Therefore, the SPP coupling strongly depends on the polarization of the incident light.

We next study the effect of the symmetry of the teeth arrangement of the 1-D grating on the SPP coupling. We rearranged the 1-D TiO_2_ grating structure into an asymmetric manor as shown in the right panel of Figure 1(a). Figure 2(d) and (e) show the *E*_**z**_ component of the *E*-field for TM and TE mode incidence, respectively. It is opposite to the case of the symmetric 1-D grating structure: the TE mode results in visible SPP coupling and the TM mode leads to nearly no SPP propagation at the TiO_2_ waveguide. Fig. S12 shows more detailed demonstration of the incident polarization dependence of the SPP coupling for the 1-D grating structures. Fig. S4 shows the physical mechanism of how the 1-D grating couples the incident light to SPP propagating along the 1-D TiO_2_ waveguide. For the symmetric grating, Fig. S4(a) shows that the direction of the E-field bends toward/away from the SiO_2_/Ag interface. This results from the local boundary condition of the electromagnetic field set by the TiO_2_ teeth structure. The negative/positive vertical component of the E-field, which is the E_z_ field in Figure 2(b), induces charge oscillations of opposite phase at the SiO_2_/Ag [51]. This constitutes the SPP excited under the 1-D grating structure that further propagates along the TiO_2_ waveguide. For the asymmetric grating, Fig. S4(b) and (c) shows the teeth structure also bends the E-field to generate finite E_z_ component at the SiO_2_/Ag interface, and induces SPP to propagate along the TiO_2_ waveguide. Comparing the Ez intensity in Figure 2(b) with that in Figure 2(e) reveals that the symmetric grating induces notably stronger Ez fields at the grating. This enhancement leads to a correspondingly stronger SPP signal along the TiO_2_ waveguide.

To search for the optimal 1-D grating structure for SPP coupling, we first varied the period **Λ** of the grating. Fig. S5 and S6 show the *|E|* ^2^ distribution at the SiO_2_/Ag interface for the symmetric and asymmetric gratings, respectively. To quantify the SPP coupling, we extracted the *|E|* ^2^ intensity line profile along the longitudinal axis of the grating structure. It is clear that the *|E|* ^2^ intensity associated to the SPP decays exponentially with distance. We estimated the SPP signal strength ***I***_**SPP*-w***_ by fitting the *|E|* ^2^ intensity line profiles with an exponential function and extrapolated the *|E|* ^2^ intensity at Y_**J**_. Figure 2(f) and (g) show ***I***_**SPP*-w***_ as a function of **Λ**, for the symmetric and asymmetric 1-D grating structures, respectively. It is clear that the optimal condition for the SPP coupling is **Λ** = 600 nm for both cases, and that the symmetric 1-D grating structure is more efficient for coupling SPP. Fig. S8 shows the incident wavelength dependence of the SPP coupling of the symmetric 1-D grating. It is clear that maximum ***I***_**SPP*-w***_ occurs to ∼793 nm wavelength, and the bandwidth is ∼14 nm.

We also fine-tuned the grating duty cycle, the TiO_2_ waveguide width ***d***_***w***_, and the teeth width ***d***_**g**_ to optimize ***I***_**SPP*-w***_. The results are summarized in Fig. S7. For the symmetric 1-D grating structure, the optimal duty cycle of the grating structure is 40 % (Fig. S7(a)), and 180 nm for the TiO_2_ waveguide width ***d***_***w***_ (Fig. S7(b)). The SPP intensity ***I***_**SPP*-w***_ basically increase with the grating tooth width ***d***_**g**_ monotonically. However, Fig. S7 shows that a local maximum occurs to *d*_***g***_ = 300 nm for *I*_**SPP*-w***_ normalized with the overall grating coupler width (2*d*_***g***_ + *d*_***w***_) (Fig. S7(c)). For the asymmetric 1-D grating structure, the optimal duty cycle is 45 % and 180 nm for the TiO_2_ waveguide width ***d***_***w***_ (Fig. S7(d) & (e)). ***I***_**SPP*-w***_ also increases with the grating tooth width ***d***_***g***_, and the normalized *I*_**SPP*-w***_ shows two local maxima, one at ***d***_***g***_ = 300 nm and the other at ***d***_***g***_ = 870 nm (Fig. S7(f)). The propagation constant of the SPP propagating along the 1-D TiO_2_ waveguide is $\beta =\left(9.95+0.066i\right)\;\mu {\text{m}}^{-1}$ , based on the simulation results shown in Figure 2(a) and Fig. S5(b), and the effective mode index *n*_eff_ = *β*/*k*_0_ = 1.26 + 0.0083*i*, where $k_{0}=2\pi /\left(793\;\text{nm}\right)$ for the incident light. Fig. S9 shows that according to the effective-index method (EIM) proposed in ref. [52], our TiO_2_ waveguide supports only single mode guiding for SPP.

**Figure 3:**
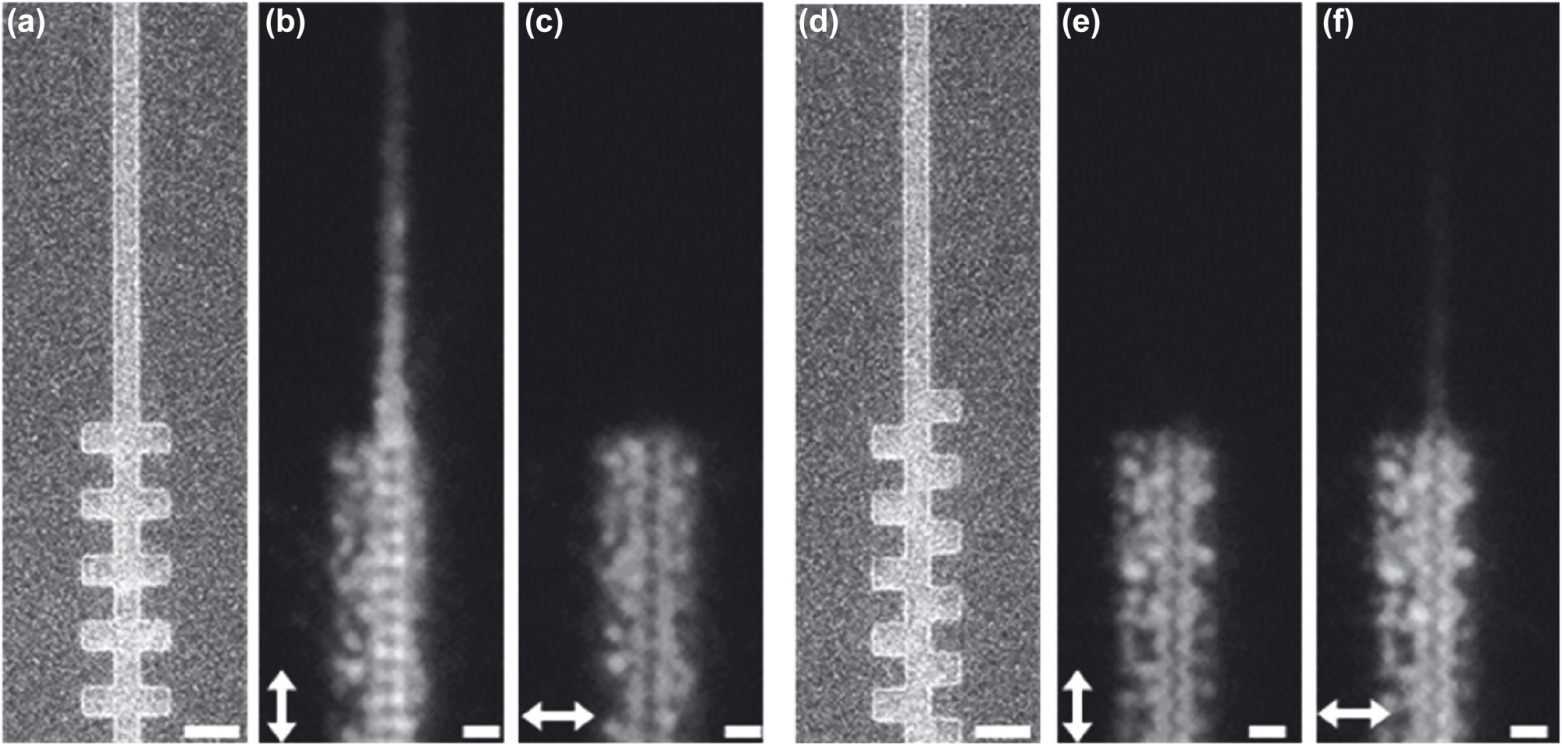
The SEM images and the up-conversion fluorescent images of the 1-D grating structures. (a) and (d) the SEM images of the symmetric and asymmetric 1-D grating structures, respectively, prior to the deposition of UNCPs. (b) and (c) the up-conversion fluorescent images of the SPP coupling with symmetric 1-D grating structure. (e) and (f) the up-conversion fluorescent images of the SPP coupling with asymmetric 1-D grating structure. The polarization of the incident light is indicated with the double arrow in each panel. The size of the scale bar is 500 nm in (a) and (d), and 1 μm in (b), (c), (e), and (f).

In accordance with the findings of the FDTD simulation, we designed and fabricated the 1-D grating structures, and characterized their performance with up-conversion fluorescence microscopy. Figure 3(a) and (d) show the SEM images of the symmetric and asymmetric 1-D grating structures, respectively, both have the same period **Λ** = 600 nm, ***d***_***w***_ = 180 nm, and ***d***_***g***_ = 300 nm. The duty cycle for the symmetric grating is 40 %, and 45 % for the asymmetric grating. Figure 3(b) and (c) show the up-conversion fluorescent images of the SPP coupling of the symmetric grating structure with the incident light polarization in the longitudinal (TM) and transverse direction (TE), respectively. It is clear that symmetric 1-D grating successfully couples the TM polarized incident light to the SPPs propagating along the TiO_2_ waveguide structure. For the case of TE polarization, the SPP coupling is not visible. For the case of the asymmetric 1-D grating structure, the SPP coupling occurs to the TE polarized light, but not for the case of the TM polarized light. The experiment results shown in Figure 3 agrees well with the simulation shown in Figure 2(a)–(e). We also varied the period of the 1-D grating structure in the experiment. Fig. S10 shows the up-conversion fluorescence images and the intensity line profiles for the symmetric grating structures. It is clear that the most intense up-conversion fluorescent intensity at the TiO_2_ waveguide occurs to the 1-D grating with period of 600 nm. To quantify the SPP coupling observed in the experiment, we extracted the intensity line profile from the images in Fig. S10 along the central longitudinal axis, and fitted the line profiles to extract the intensity at ***Y***_***J***_. The results are shown in Figure 2(f), and it clear that the symmetric grating with period 600 nm is the optimal, which agrees qualitatively with the simulation. For the case of asymmetric 1-D grating, Fig. S11 indicates the optimal SPP coupling also occurs to the period of 600 nm, and Figure 2(g) shows the consistency between the experiment and the simulation.

With Fig. S13 we show the experimental results of how SPP coupling depends significantly on the polarization of the incident light. The strongest coupling occurs to the TM incident light for the symmetric 1-D grating structure, and TE for the asymmetric grating structure. This agrees well with simulation shown in Fig. S12. Note that most of the SPP coupling devices are limited to couple the incident light polarized in the direction parallel to the SPP propagation (TM mode), due to the directionality of charge oscillation. Here we demonstrate that our asymmetric 1-D grating structure enables the coupling of the TE mode incident light to SPPs propagating along the TiO_2_ waveguide structure.

We proceeded to estimate the coupling efficiency of the 1-D grating structures based on the time-averaged Poynting vector distributions obtained from the simulation of SPP coupling. Fig. S14(a) shows the distribution of the *y*-component of the time-averaged Poynting vector of the SPP propagating at the SiO_2_/Ag interface under the symmetric 1-D grating with period **Λ** = 600 nm. This clearly shows that the 1-D grating transferred the energy flux of the incident light to the SPPs propagating upward along the TiO_2_ waveguide. Fig. S14(b) shows the cross-sectional distribution of the longitudinal component *S*_**y**_ of the time-averaged Poynting vector at *y* = 24.6 μm in Figure 2(a). Due to the negative real part of the dielectric constant of Ag in the optical wavelength range, the time-averaged Poynting vector of SPP under the SiO_2_/Ag interface, i.e. the Ag film region, points to the direction opposite to that of SPP propagation [53], [54], [55]. For simplicity, we numerically integrating |*S*_**y**_| over the cross-sectional plane to obtain the power $P_{\text{SPP}}\left(y\right)$ at *y* = 24.6 μm. We then repeated the calculation for $P_{\text{SPP}}\left(y\right)$ at different positions (*y*-coordinates) along the TiO_2_ waveguide, and extrapolate the ***P***_**SPP*-w***_ at ***Y***_***J***_ **,** according to an exponential-curve fitting to $P_{\text{SPP}}\left(y\right)$ . The SPP coupling efficiency ***Q*** of the 1-D grating is defined as the ratio of the power of incident light *P*_*in*_ to the power of the SPP propagation along the TiO_2_ waveguide ***P***_**SPP*-w***_, i.e.

(1) $$Q=\frac{P_{\text{SPP }-\text{ w}}}{P_{in}}.$$

We repeat the procedure described above for 1-D grating with different periods. Fig. S14 (c) and (d) show that the optimum efficiency occurs to the period of 600 nm, for both type of grating structure. The optimum efficiency for the symmetric grating structure is 19.1 %, and 1.7 % for the asymmetric grating structure.

Due to our limited knowledge of the absolute up-conversion fluorescent efficiency of the UCNPs, it is not feasible in the experiment to measure the SPP coupling efficiency of the 1-D gratings with the upconversion fluorescence microscopy. Instead, we adapt the dual-grating approach reported in ref. [24], where two identical SPP coupling structures were exploited for the coupling efficiency measurement of our 1-D grating. Fig. S15(a) shows the schematics of the sample design, which contains two identical 40-period 1-D grating with **Λ** = 600 nm. Both grating was connected with a TiO_2_ waveguide, and the length of the TiO_2_ waveguide was varied from 2 μm to 20 μm. The sample was fabricated with the same procedure described above, and for consistency, a layer of UCNPs was also spin-coated on top of the sample. In the experiment, one of the 1-D grating, which we referred to as “the coupling grating”, was illuminated with 793-nm-wavelength incident light with the same optical system shown in Fig. S2. For the imaging system, we removed the bandpass filter (F in Fig. S1) in order for the CCD camera to record the sample image with 793-nm-wavelength light from the sample, the upconversion fluorescent signal intensity is orders of magnitude smaller than that of the 793-nm-wavelength signals, therefore, is negligible in the measurement. Fig. S15(b) shows a typical 793-nm-wavelength image of the device with the incident light illuminating only the coupling grating. We then extracted the intensity line profiles along the longitudinal axis of the device structure in the images. Fig. S15(d) shows the line profile extracted from the image in Fig. S15(b). We summed the intensities over the range of the second grating, which we referred to as “decoupling grating”, and plotted the results as a function of the TiO_2_ waveguide length of each device. Fig. S15(e) shows the TiO_2_ waveguide lengths dependence of the 793-nm-wavelength intensities collected from the decoupling grating. The exponential fitting results in a decay length of 14.1 μm for the SPP signal propagating along the TiO_2_ waveguide. The intensity of the incident light was characterized by illuminating the incident light at a blank area on the sample, and the image of the incident light reflected by the plane Ag film was shown in Fig. S15(c). Taking the total intensity of the illuminated area in Fig. S15(c) as proportional to the power of the incident light *P*_in_, and similarly that of the 1-D decoupling grating as proportional to the output power of the 793-nm-wavelength light *P*_out_. Following ref. [24], we have

(2) $$P_{\text{out}}=P_{in}\cdot \eta \cdot e^{-d/\tau }\cdot \eta ,$$

where *η* is the efficiency of the 1-D grating coupling light into SPP and vice versa, *d* is the length of the TiO_2_ waveguide, and *τ* decay length of SPP propagating through the TiO_2_ waveguide. From eq.(1) we estimated the coupling efficiency *η* for each device. The results are shown in Fig. S15(f). The coupling efficiency of our 40-period 1-D grating is ∼13 % for the systems with 2-um, 5-um, and 10-um TiO_2_ waveguide structures, and slightly decreases to 12 % for that with 20-um TiO_2_ waveguide structure. The results are somewhat lower than the estimation from numerical simulation results. This may result from the difference between the structure of the real 1-D grating fabricated with limited precision and that in the simulation model constructed with simple and ideal geometry.

Regarding the performance of the SPP coupling of the devices reported in the literature, we would like to point out that both the coupling efficiency of a coupler and its foot-print size should be equally critical for the advance of plasmonics. Therefore, we define the figure of merit (FOM) of an SPP coupler as

(3) FOM=η/A,

where *η* is the coupling efficiency of the SPP coupler and ***A*** the area of its footprint. Fig. S16 and Table S1 show the FOM as a function of ***A*** for various SPP couplers that have been reported in the literature. It is clear that the performance of our 1-D grating is quite competitive to most of the SPP couplers. We would like to point out that, for the cases of the top two FOMs, the SPP couplers indeed couple the incident light to 2-D SPPs. For applications, further device construction would be needed to facilitate the coupling the 2-D SPP to TiO_2_ waveguides. Therefore, our 1-D grating has the highest coupling efficiency for coupling incident light to the 1-D SPP along the TiO_2_ waveguide.

**Figure 4:**
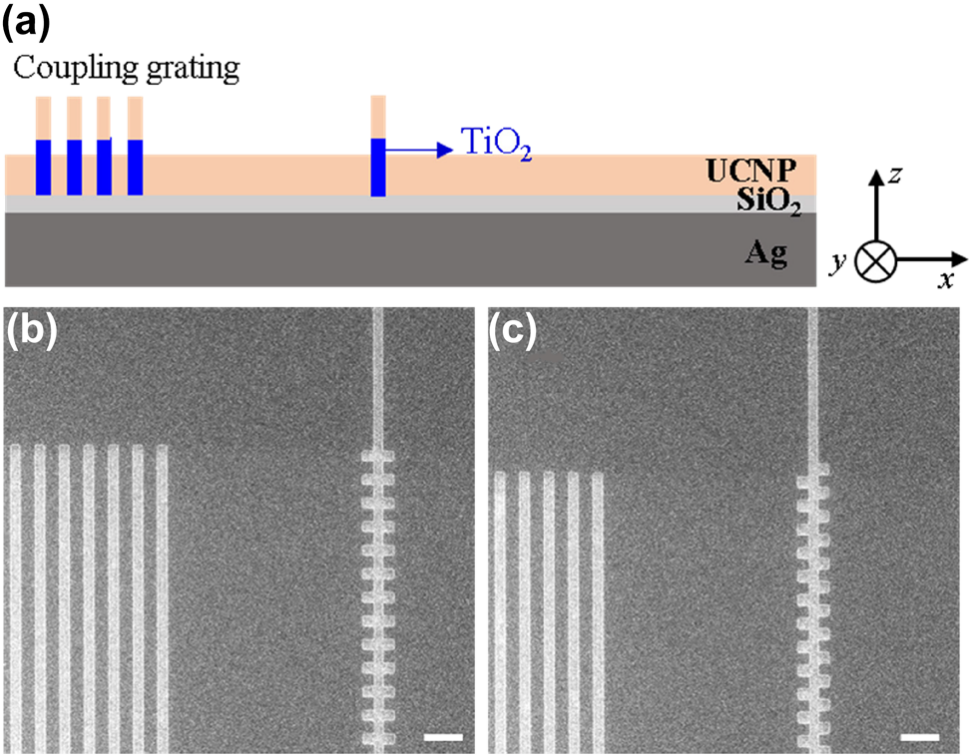
The schematics of the routing of the SPP wave with the 1-D TiO_2_ grating structure. (a) The cross-sectional view of the sample design. The sample design consists of a coupling grating on the left, which couples the incident light to SPP at SiO_2_/Ag interface and the 1-D TiO_2_ grating structure on the right. The thickness of each layer and the height of the TiO_2_ structure are the same as those in Figure 1. (b), (c) SEM images of the SPP grating coupler and the symmetric and asymmetric 1-D grating structure prior to the coating of UCNPs, respectively. The gating period is **Λ** = 600 nm, the grating teeth width ***d***_***g***_ = 300 nm, the TiO_2_ waveguide width ***d***_***w***_ = 180 nm. The size of the scale bar is 1 μm.

We next demonstrate the routing of SPPs with the 1-D grating structures, i.e. coupling of 2D-SPPs to SPPs propagating along the TiO_2_ waveguide. The scheme for plasmonic routing demonstration incorporates a linear array grating coupler on the left of a 1-D grating structure to produce 2-D SPP waves in the first place. Figure 4(a) show the cross-sectional view of the sample design. The SEM images in Figure 4(b) and (c) present a top-view of the linear array grating couplers and the symmetric and asymmetric 1-D grating structures, respectively.

**Figure 5:**
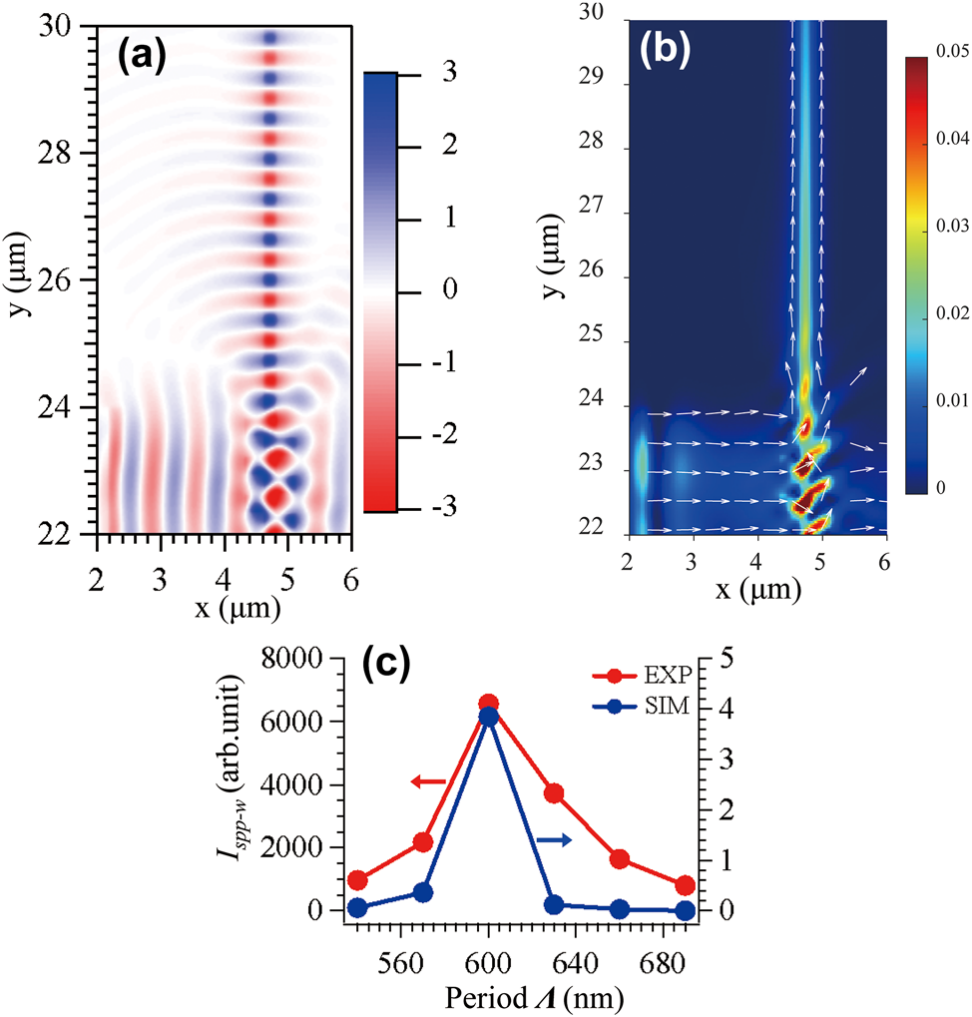
The simulated SPP routing with the asymmetric grating structure. (a) A snap shot of the simulated *E*_**z**_-component of the *E*-field distribution at SiO_2_/Ag interface, and (b) the time-averaged distribution of the Poynting vector associated to the SPPs. The arrows indicate the local direction of the Poynting vector. (c) The simulated (blue circles) and the experimental (red circles) routed SPP intensity at the waveguide region as a function of asymmetric grating period **Λ**.

We started with the optimal symmetric and asymmetric grating structures: the grating period **Λ** = 600 nm, the grating tooth width ***d***_***g***_ = 300 nm, and the TiO_2_ waveguide width ***d***_***w***_ = 180 nm. Figure 5(a) show the simulated *E*_**z**_-component of the *E*-field distribution at the SiO_2_/Ag interface for the asymmetric 1-D grating structure. The linear array grating (not included in the panel) couples the incident light polarized in the *x*-direction to the 2-D SPPs travelling toward the asymmetric 1-D grating, with the wave-front parallel to the *y*-axis. When reaching the asymmetric 1-D grating, the TiO_2_ teeth breaks the E_z_-field of the SPP into a periodical pattern with alternating directions (up and down). This implies charges oscillations induced by the local field similar to that indicated in Figure 2(b) and (e) and Fig. S4 occurs, thus initiates the 1D-SPP wave to propagation along the TiO_2_ waveguide. The local horizontal wave-fronts emerging from the grating structures also signatures the SPPs propagate along the TiO_2_ waveguide. Similar E_z_ wave front breaking with the TiO_2_ teeth of the symmetric grating can also seen in Fig. S17(a).

To confirm the routing in the simulation in terms of energy flow, we calculated the time-averaged Poynting vector at the SiO_2_/Ag interface. The results are shown in Figure 5(b), and it is clear that the energy flux of the 2-D SPP initially aims toward the grating, and then turns toward the TiO_2_ waveguide at the 1-D grating structure. The asymmetric 1-D grating routed the SPPs with a 90° turn in the propagation direction. Fig. S17 shows similar results for the routing of SPPs with symmetric 1-D grating structure, however, the magnitude of the Poynting vector along the TiO_2_ waveguide structure after the routing is seemingly smaller than that of the case of the asymmetric 1-D grating structure. For clarity, Fig. S18 shows the simulated *E*_**z**_-component with a larger field of view to include both the linear grating coupler and the 1-D grating structures.

**Figure 6:**
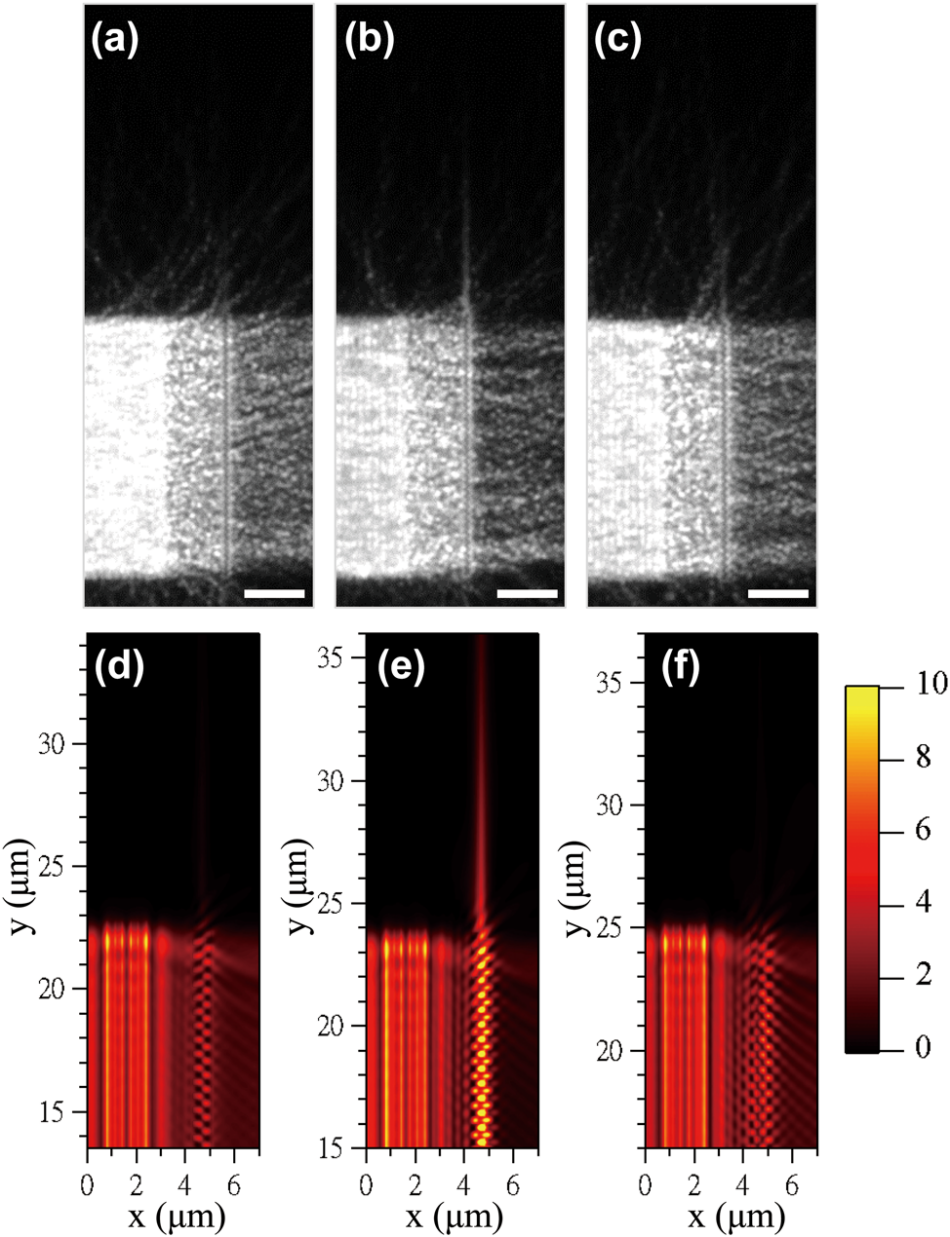
Experimental and simulation results of SPP routing with asymmetric 1-D grating. (a), (b), and (c) The up-conversion fluorescent images of the SPP routing with the asymmetric grating structures with the grating period of 570 nm, 600 nm, and 630 nm, respectively. (d), (e), and (f) The simulated *E*-field intensity distribution *|E|* ^2^ of the SPP routing with the asymmetric grating structure with the grating period of 570 nm, 600 nm, and 630 nm, respectively. The size of the scale bar in (a)–(c) is 5 μm.

Next, we investigated the period dependence of the SPP routing of the 1-D grating structures in both experiment and simulation. Figure 6(a)–(c) shows the up-conversion fluorescent images of the SPP routing with the asymmetric 1-D grating structures of different periods, and Figure 6(d)–(f) shows the corresponding *|E|* ^2^ intensity distribution at the SiO_2_/Ag interface from the simulation for comparison. It is clear that the routing of SPP can be observed most clearly for the case of the grating with period of 600 nm in both the simulation and the experiment. To quantify the period **Λ** dependence of SPP routing, we extracted the intensity line profiles from both the experimental and the simulation images, fitted the data with an exponential function and extrapolated the intensity ***I***_**SPP*-w***_ at ***Y***_***J***_. Figure 5(c) shows ***I***_**SPP*-w***_ as a function of the grating period **Λ**. Both the experiment and the simulation show consistent trends with the optimum occurs to the case of **Λ** = 600 nm. Fig. S19 shows the simulated incident-light wavelength dependence of the routing, the bandwidth of the central peak at 793 nm wavelength is also ∼14 nm. Fig. S20 shows the intensity line profile associated to the routed SPP in the TiO_2_ waveguide. The decay length τ is ∼8.37 μm for the simulation and ∼7.3 μm for the experiment, which corresponds to the propagation loss $\left(4.343\times \tau^{-1}\right)$ of 0.52 μm^−1^ and 0.59 μm^−1^, respectively.

For symmetric 1-D grating structures, Fig. S21 shows the comparison between the experiment and the simulation of the grating period **Λ** dependence of the SPP routing. Fig. S21(c) indicates the optimum period for SPP routing also occurs to the case of **Λ** = 600 nm. However, the intensity of the routed SPP by the symmetric 1-D grating is nearly half of that by the asymmetric 1-D grating.

We estimated the routing efficiency of the 1-D grating structure in the simulation. The efficiency of the SPP routing is defined as the ratio of the power of the 1-D SPP at ***Y***_***J***_ to that of the incident 2-D SPP. Fig. S22(a) shows the *x*-component *S*_**x**_ of the time-averaged Poynting vector of the incident 2-D SPPs at the SiO_2_/Ag interface, and Fig. S22(c) a cross-sectional view of *S*_**x**_ in the y-z plane at *x* = 4 μm, where the 2-D SPP is presumably reaching the boundary of the 1-D grating. The 1-D grating was absent in the simulation of Fig. S22(a) and (c) to eliminate the effect of the reflection of the 2D SPP from the 1-D grating. The power of incident the 2-D SPPs is calculated by numerically integrating the magnitude of |*S*_**x**_| shown in Fig. S22(a). For the routed SPP, Fig. S22(b) shows the *S*_**y**_ component of the time-averaged Poynting of the routed SPP at the SiO_2_/Ag interface, and Fig. S22(d) the typical distribution of *S*_**y**_ at x-z cross-sectional plane. For the power of 1-D SPP at ***Y***_***J***_, we followed the aforementioned procedure to obtained the magnitude of ***P***_**SPP*-w***_. Fig S23 (a) and (b) shows the period dependence of the SPP routing efficiency, and the maximum SPP routing efficiency occurs to the case of **Λ** = 600 nm with 5.7 % and 4.0 % efficiency for the asymmetric and symmetric grating structures, respectively. Note that for routing the 2-D SPPs into 1-D SPP along the TiO_2_ waveguide, the asymmetric gratings are more effective than the symmetric grating structures.

We also carried out experiment to estimate the routing efficiency of the optimal asymmetric 1-D grating with **Λ** = 600 nm. We follow the scheme similar to that presented in Fig. S22. Due to the evanescent nature of the SPP field, we resorted to the upconversion fluorescence microscopy for the measurement. Fig. S24(a) and (b) show the upconversion fluorescent images of 2-D SPP propagating on the SiO_2_/Ag. In Fig. S24(a) the linear array grating couples the incident light to the 2-D SPP propagating on blank SiO_2_/Ag interface. In Fig. S24(b) the 2-D SPP passes the 1-D grating of interest. However, the grating structure results in different nearfield strength of the SPP compared to that associated to the 2-D SPP at plane SiO_2_/Ag interfaces, and the upconversion fluorescent signal was obviously more enhanced at the linear array grating, 1-D grating and the TiO_2_ waveguide compared to that of the planar region. To factor out the effect of the intrinsic difference in the nearfield distributions on the up-conversion fluorescent signals, we opt to exploit the fluorescent signals from the 2-D SPPs to estimate the upper limit of the SPP routing efficiency of the 1-D grating. We first extracted the intensity line profiles from the images in Fig. S24(a) and (b), and normalize each line profile with the intensity at the right edge of the linear array grating, which corresponds to the initial intensity of the 2-D SPP leaving the grating. Fig. S24(c) shows the resulting line profiles. The line profile of the 2-D SPP passing the asymmetric 1-D grating shows a stepwise drop of the SPP intensity after passing the grating structure. By taking the ratio of the SPP intensity from the line profiles at the grating position, we can define the transmission T for the case of the 2-D SPP passing the 1-D grating. i.e.

(4) $$T=\frac{I_{G}}{I_{B}},$$

where *I*_*G*_ is the line profile intensity at the grating structure retrofitted from the data after the grating structure, *I*_*B*_ is the line profile intensity of the blank SPP image estimated at a distance from the linear grating equivalent to that between the linear gating and the 1-D grating based on an exponential fitting to the profile. The upper limit of the routing efficiency *R*_max_ can be obtained by *R*_max_ = 1 − *T*, which is 15 % ± 2 % from the measurement. This upper limit of the routing efficiency is higher than the numerical calculations, since other mechanisms, such SPP-light coupling at the 1-D grating, will also contribute to the loss of the SPP signals. The results shown above demonstrate that our 1-D grating structures can both couple the incident light or 2-D SPPs into 1-D SPPs as a coupler or a router, respectively. Thus, our 1-D grating structure is a promising design that offers a versatile function for the integration of plasmonic devices.

## 4 Conclusions

We demonstrated with both simulation and experiment the coupling and routing of SPPs at the SiO_2_/Ag interface with 1-D symmetric and asymmetric grating structures. The experimental results agree with the simulation results qualitatively. A 1-D symmetric grating structure with a period of 600 nm was used to create constructive interference of the SPP waves to propagate alone the TiO_2_ waveguide with the optimal efficiency of ∼13 % for the SPPs. Furthermore, our 1-D grating structures can also serve as the SPP router, allowing the coupling of 2-D SPP waves to 1-D TiO_2_ waveguide systems. The routing efficiency of the asymmetric grating structure for SPP is superior to that of the symmetric grating structure. Furthermore, the strong dependence of the light-SPP coupling efficiency of the 1-D grating structures on the polarization of the incident light allows further development for light polarization selection /detection devices. Therefore, our 1-D grating design offers a flexible framework for coupling and manipulating SPPs propagating at the SiO_2_/Ag interface.

## Supplementary Material

Supplementary Material Details
