# Supplementary material for: One-dimensional dielectric grating structure for plasmonic coupling and routing
Source: Nanophotonics. 2025 Dec 5;14(27):5577–88. doi: 10.1515/nanoph-2025-0506 (PMC12717937; doi:10.1515/nanoph-2025-0506)
Supplement: Supplementary file 1 — Supplementary Material Details [file j_nanoph-2025-0506_suppl_001.pdf]

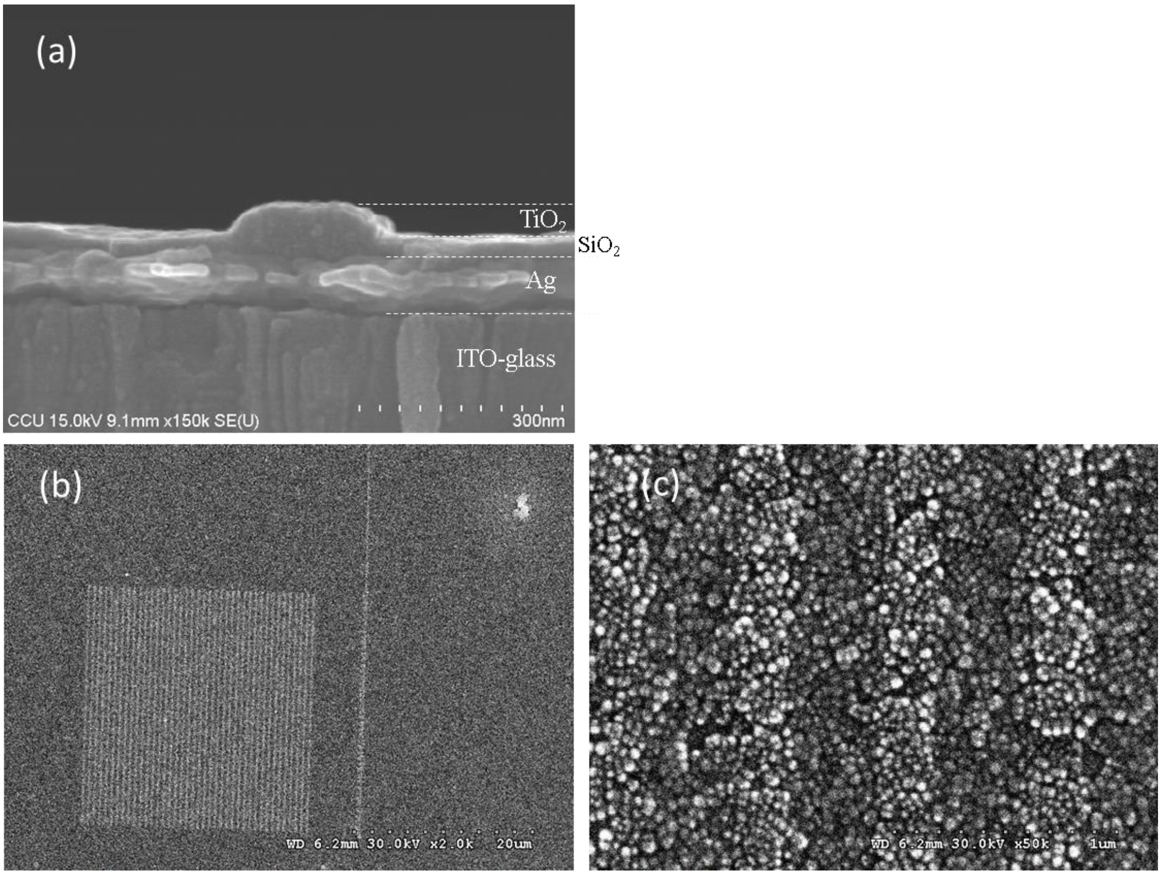

Fig. S1 (a) Field-emission scanning electron microscope images of the cross-sectional view of the device in Fig. 1 before the spin-coating of the UCNPs. The horizontal dashed line indicates the interfaces between layers of different materials as labelled. (b) and the (c) The SEM image of the distribution of the UCNPs spin-coated on top of the sample. (b) shows the overall view of the linear grating, the 1-D grating and the SPP waveguide. (c) shows the enlarged view of the area within the linear grating.

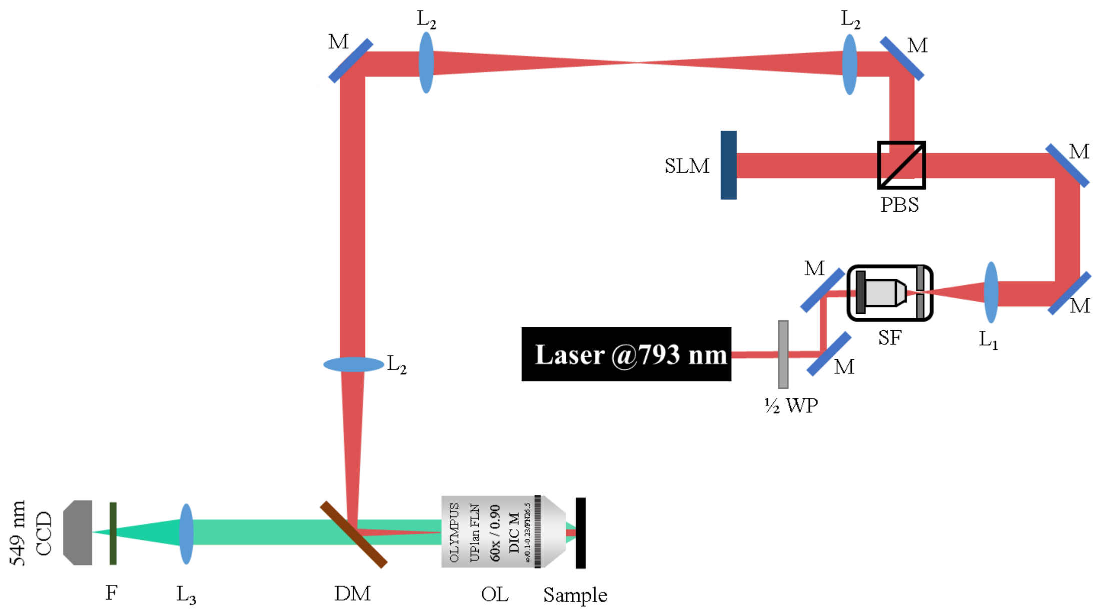

Figure S1(2). The schematics of the experimental setup of the up-conversion fluorescent microscopy. The list of optical components labeled in the schematics follows.

½WP: ½ waveplate (800 nm)

M: Mirror

SF: Spatial Filter

L1: Lens (f = 100 mm)

PBS: Polarizing Beam-splitter

SLM: Spatial Light Modulator

L2: Lens (f = 300 mm)

DM: Dichroic Mirror (650 nm short-pass) (>50%)

OL: Objective Lens (60x / NA 0.9)

L3: Lens (f = 200 mm)

F: Bandpass Filter (549.0/16.7 nm) (>95%).

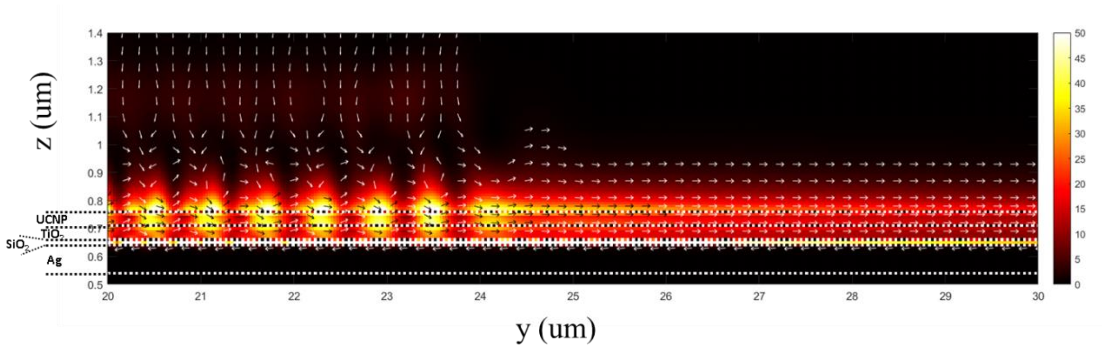

Fig. S3 Simulated time-average Poynting vector distribution associated to the results shown in Fig. 2(a). The color map shows the distribution of the $|E|^2$ intensity, and the arrows (white and black) shows the direction of the time-averaged Poynting vector. For clarity, all arrows are drawn with equal length, and their colors are chosen to contrast with the background.

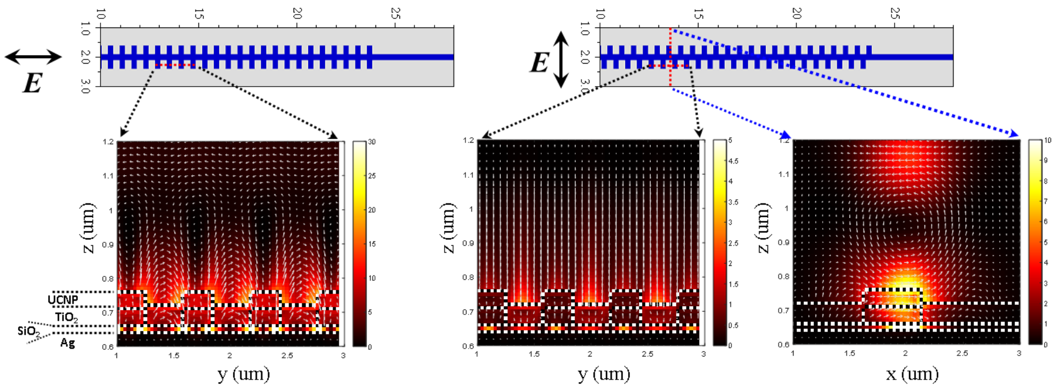

(a) (b) (c)

Fig. S4 Snapshot of E-field distribution of (a) 1-D symmetric grating illuminated with TM mode incident light. (b) and (c) the 1-D asymmetric grating illuminated with TE mode incident light. The red dashed line segments in the schematics of the 1-D gratings on the top indicates the location where the E-field distributions in the y-z or x-z plane was extracted. The color map in each panel indicate the $|E|^2$ the E-field (here $|E|^2 = E_y^2 + E_z^2$ for (a) and (b), and $|E|^2 = E_x^2 + E_z^2$ for (c)) , and the arrows indicate the direction of the E-field projected on the plane of cross-section. The turning of the E-field direction toward or away from the SiO_2/Ag interface results in the temporal fluctuation of the conduction electrons, thus the excitation of SPP at the interface.

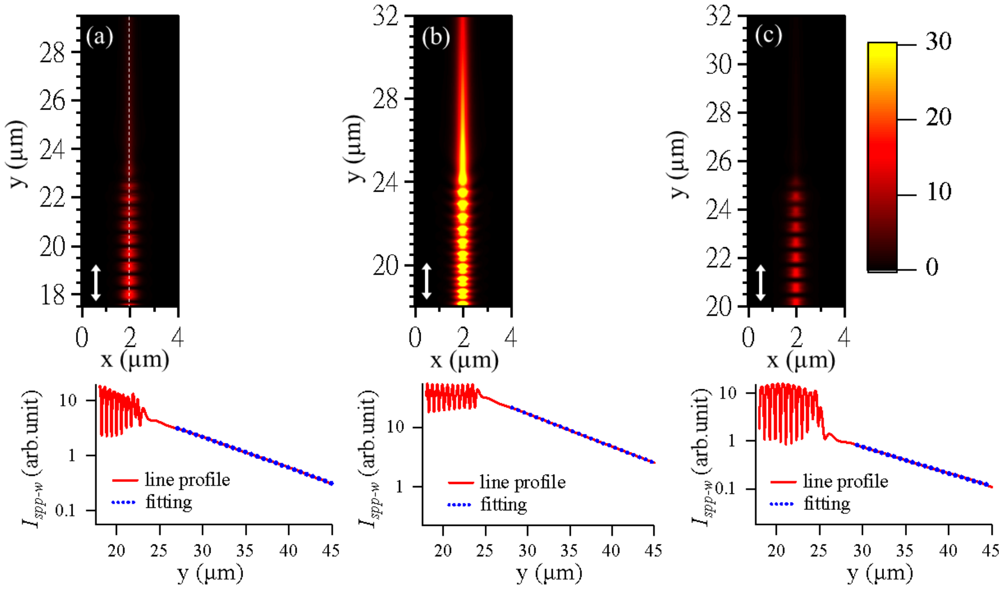

Fig. S5 The simulated period Λ dependence of the SPP coupling of the symmetric 1-D grating structure. The upper panels in (a), (b), and (c) show the simulated *E*-field intensity $|E|^2$ distribution at the SiO_2/Ag interface under the grating structures, with the periods of 570 nm, 600 nm, and 630 nm, respectively. The bottom panels show the longitudinal intensity line profiles (solid curves) extracted from the center of the structure, as indicated with the white dash line in (a). The dashed lines are the results of curve-fitting with the function of $y = y_0 + Ae^{\left(-\frac{Y_J - x_0}{\tau}\right)}$, τ is 7.66±0.01 μm, which corresponds to the propagation loss (4.343× τ^{-1}) of 5.67 um^{-1}. This corresponds to an imaginary component of the complex propagation constant, $\beta = \beta_R + i\beta_I$, of the SPP propagating along the 1-D TiO_2 waveguide, with $\beta_I = 1/2\tau = 0.066\ (\mu m^{-1})$. The polarization of the incident light is indicated with the double arrows in each panel.

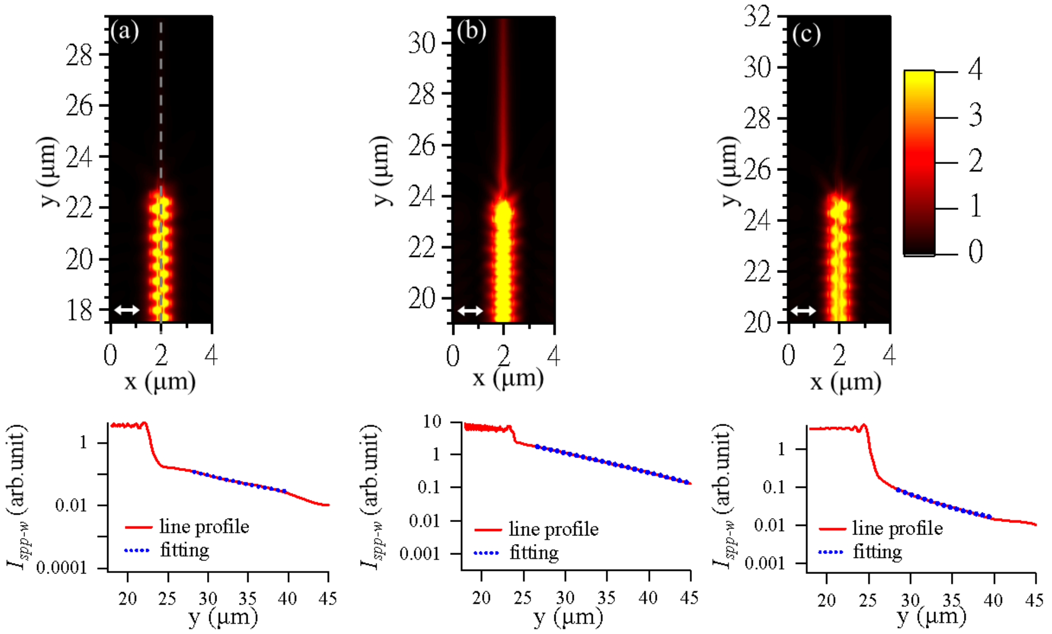

Fig. S6 The simulated period Λ dependence of the SPP coupling with the asymmetric 1-D grating structures. The top panels in (a), (b), and (c) show the simulated E-field intensity $|E|^2$ distribution at the SiO_2/Ag interface under the grating structures with the periods of 570 nm, 600 nm, and 630 nm, respectively. The bottom panels show the longitudinal intensity line profiles (solid curves) extracted at the center as indicated with the white dash line in (a). The curve-fitting analysis (dashed curves) follow the approach described in Fig. S5.

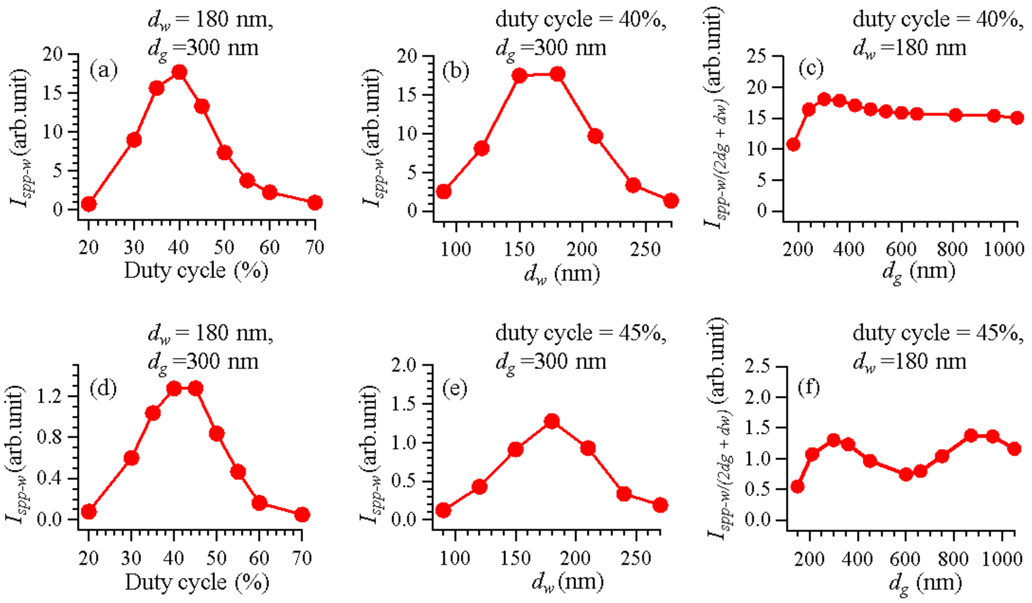

Fig. S7 (a)-(c) the simulated structural dependence of the coupled SPP intensity $\boldsymbol{I_{SPP,w}}$ at the TiO_2 waveguide and the symmetric 1-D grating structures, and (d)-(f) those for the asymmetric 1-D grating structures. (a) and (d) the SPP intensity at the TiO_2 waveguide as a function of the grating duty cycle. (b) and (e) the SPP intensity at the TiO_2 waveguide as a function of the TiO_2 waveguide width $\boldsymbol{d_w}$. (c) and (f) the SPP intensity at the TiO_2 waveguide normalized with the total grating coupler width ($\boldsymbol{2d_g}$+ $\boldsymbol{d_w}$) of the grating structure as a function of the grating tooth width $\boldsymbol{d_g}$. The controlled structural parameters are indicated in each panel.

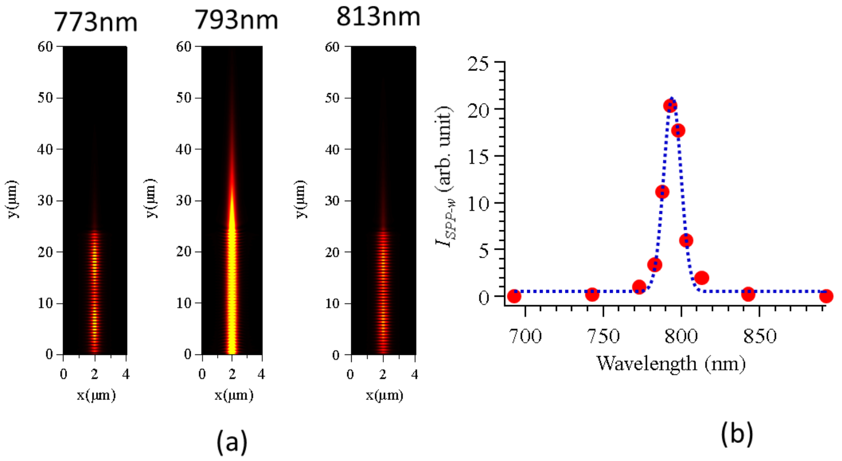

Fig. S8 The simulated incident light wavelength dependence of the SPP coupling for the optimal symmetric 1-D grating structure. (a) The E-field intensity $|E|^2$ for incident light as labelled. (b) the simulated (filled circle) SPP intensity at the TiO_2 waveguide as a function of the wavelength. The solid curve represents the result of a Gaussian fit to the data. The bandwidth according to the fitting parameter is ~14 nm.

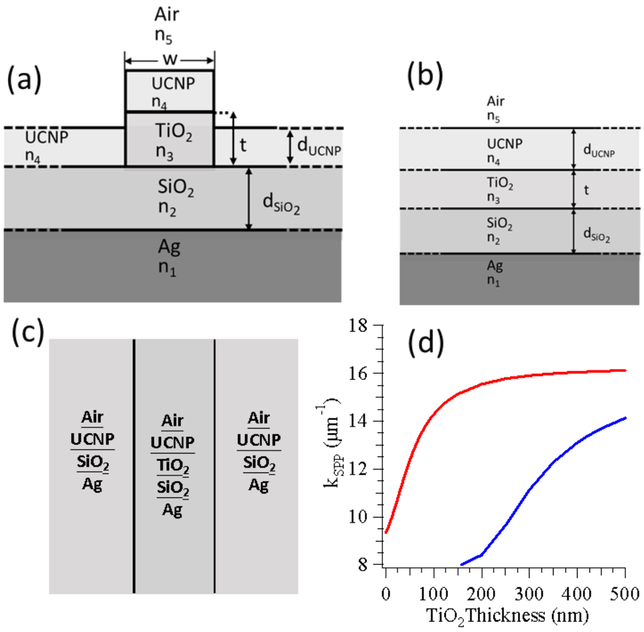

Fig. S9 The effective-index model (EIM) of the TiO_2 waveguide[1]. (a) the cross-sectional view of the 1-D SPP waveguide shown in Fig. (b), where the width and the thickness of the TiO_2 waveguide is $w = 180\ nm$, and $t = 40\ nm$, respectively, the thickness of UCNP layer $d_{UCNP} = 50\ nm$, and the thickness of the SiO_2 layer $d_{Sio2} = 20nm$. (b) the 5-layer and (c) the 3-layer structures corresponding to the first and the second steps of the EIM model. (d) The dispersion curves of the SPP waveguide mode associated to the 5-layer model in (b). It is clear that TiO_2 waveguide with $t = 40\ nm$ is a single-mode waveguide for the 5-layer structure model. Furthermore, for the 3-layer model in (c), the condition for the cutoff width w for single mode waveguide is

$$w_{min} = \pi / \sqrt{k_{SPP}^2 - k_{SPP0}^2}$$

, were $k_{SPP}$is the SPP wavevector for the 5-layer model, and $k_{SPP0}$the SPP wavevector of the 4-layer structure outside of the waveguide, i.e. Air/UCNP/SiO_2/Ag. For 793-nm-wavelength light, the real part of $k_{SPP} = 2\pi/536 \cdot nm^{-1}$[2] and the real part of $k_{SPP0} = 2\pi/675 \cdot nm^{-1}$[3]. This results in $w_{min} = 441nm$. Therefore, our 180-nm width TiO_2 SPP waveguide is a single –mode waveguide according to the EIM model.

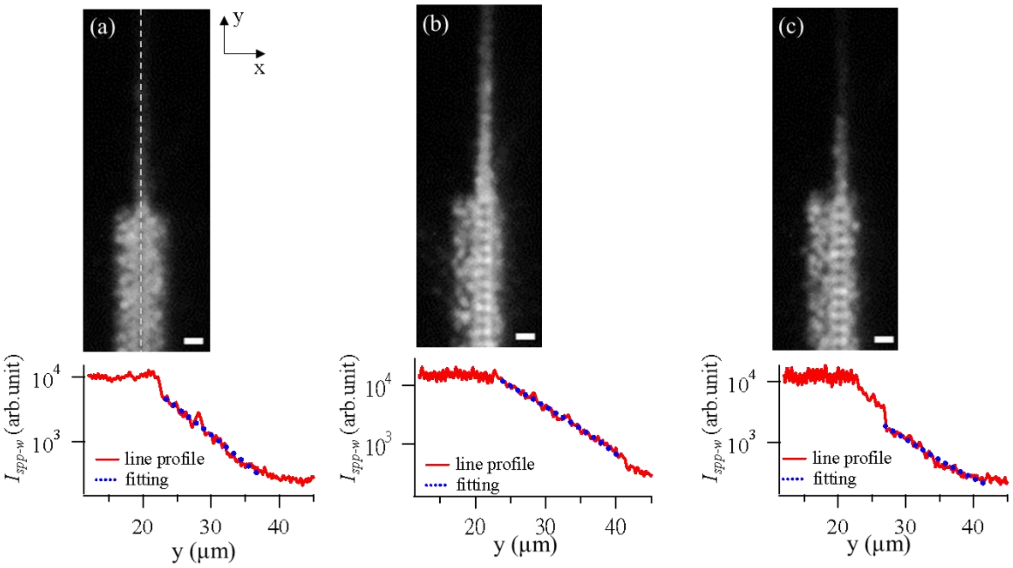

Fig. S10 Measurement of the period Λ dependence of the SPP coupling with symmetric 1-D grating structure. The top panels in (a), (b), and (c) show the up-conversion fluorescent images of the SPP coupling with symmetric 1-D grating structure with the grating periods of 570 nm, 600 nm, and 630 nm, respectively. The bottom panels are the longitudinal fluorescent intensity line profiles (solid curves) extracted from the center of the grating structure as indicated with the white dash line in (a). The curve-fitting analysis (dashed curves) follows the approach described in Fig. S5.

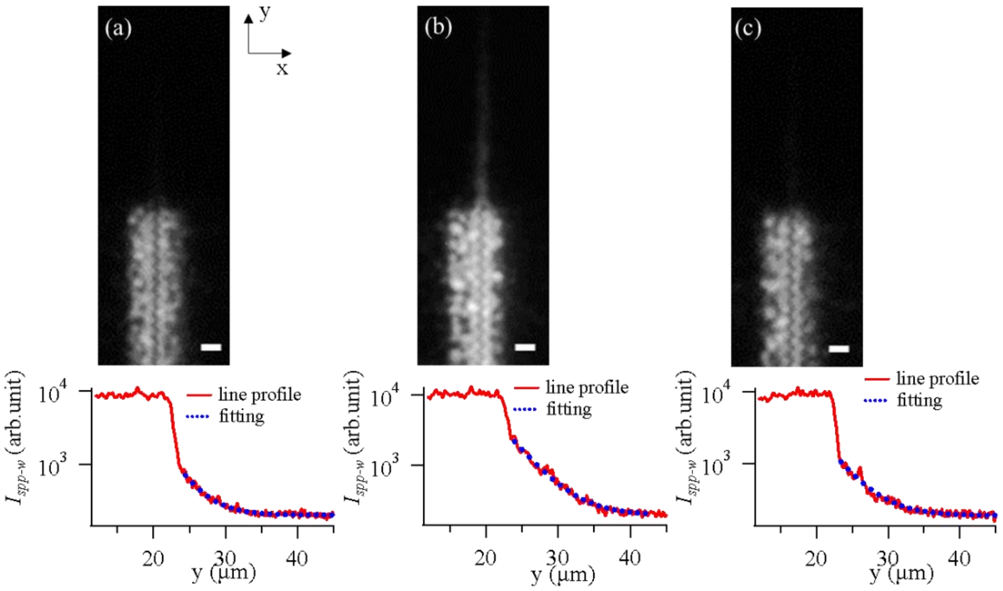

Fig. S11 Measurement of the period Λ dependence of the SPP coupling with asymmetric 1-D grating structures. The top panels in (a), (b), and (c) show the up-conversion fluorescent images of the SPP coupling with symmetric 1-D grating structure with the grating periods of 570 nm, 600 nm, and 630 nm, respectively. The bottom panels are the longitudinal fluorescent intensity line profiles (solid curves) extracted from the center of the grating structure as indicated with the white dash line in (a). The curve-fitting analysis (dashed curves) follows the approach described in Fig. S5.

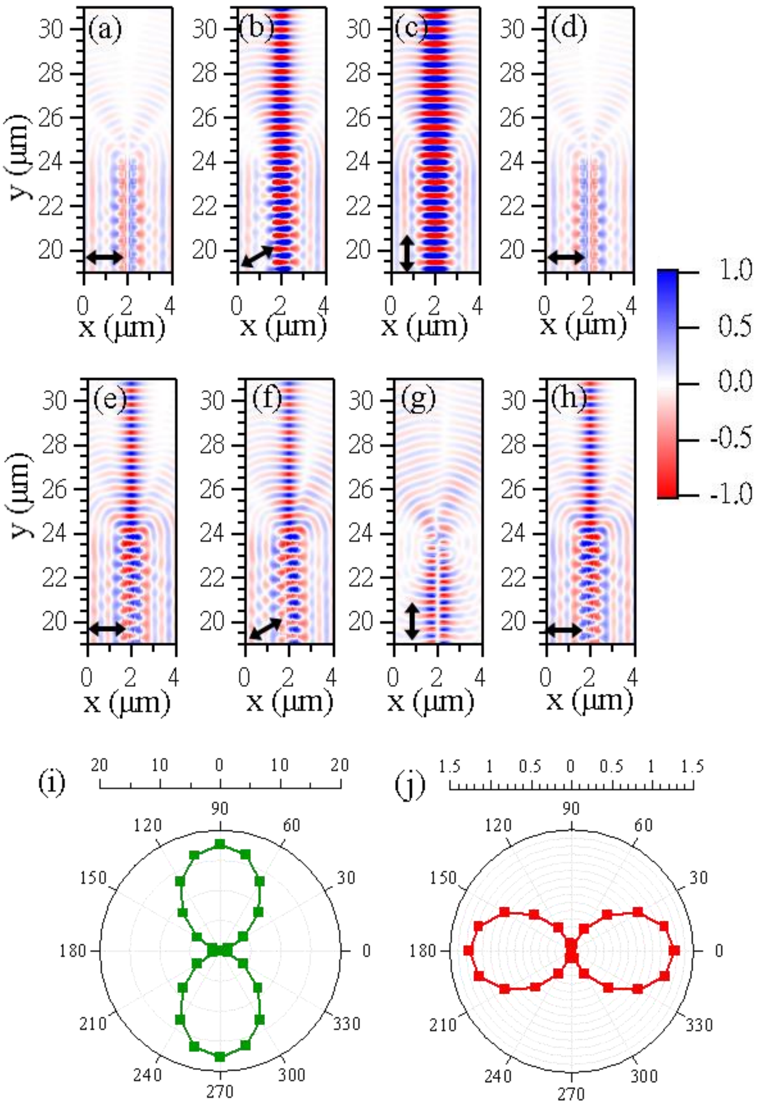

Fig. S12 (a) The simulated incident light polarization dependence of the SPP coupling of the 1-D grating structures. (a)-(d) show the E_z-component of E-field distribution at the SiO_2/Ag interface under the symmetric 1-D grating structure and (e)- (h) show that of the asymmetric 1-D grating structure. The incident light polarizations are indicated by the double arrows in each panel. (i) and (j) The polar plots of the simulated SPP intensity at the TiO_2 waveguide connected to the symmetric grating structure, and the asymmetric grating structure, respectively. The symmetric and asymmetric 1-D grating structures both have the same period $\boldsymbol{\Lambda}$ = 600 nm, $\boldsymbol{d_w}$ = 180 nm, and $\boldsymbol{d_g}$ = 300 nm. The duty cycle for the symmetric grating is 40%, and 45% for the asymmetric grating.

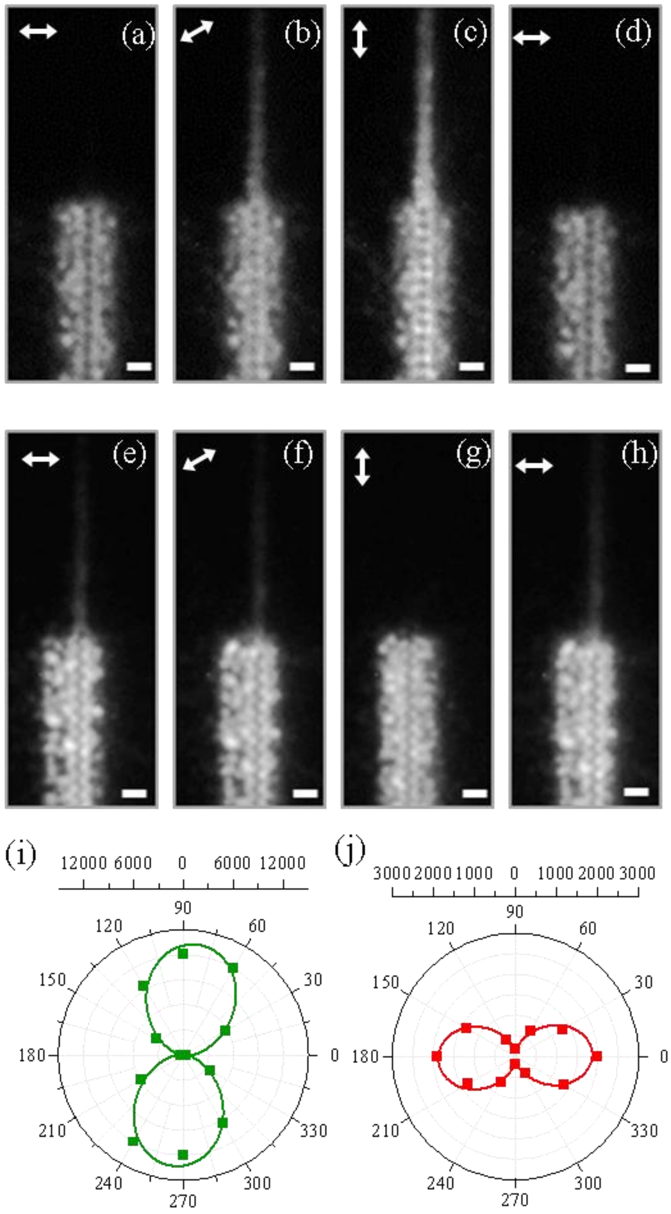

Figure S13 Measured incident light polarization dependence of the SPP coupling with 1-D grating structures. (a)-(d) The fluorescent images of the SPP coupling with the symmetric grating structure and (e)- (h) show those of the asymmetric grating structure. The orientations of the incident light polarization are indicated with the double arrows in each panel. (i) and (j) The polar plots of the fluorescent SPP intensity as a function of the polarization angle for the SPP coupling of the symmetric grating structure, and the asymmetric grating structure, respectively. The symmetric and asymmetric 1-D grating structures both have the same period $\boldsymbol{\Lambda}$ = 600 nm, $\boldsymbol{d_w}$ = 180 nm, and $\boldsymbol{d_g}$ = 300 nm. The duty cycle for the symmetric grating is 40%, and 45% for the asymmetric grating.

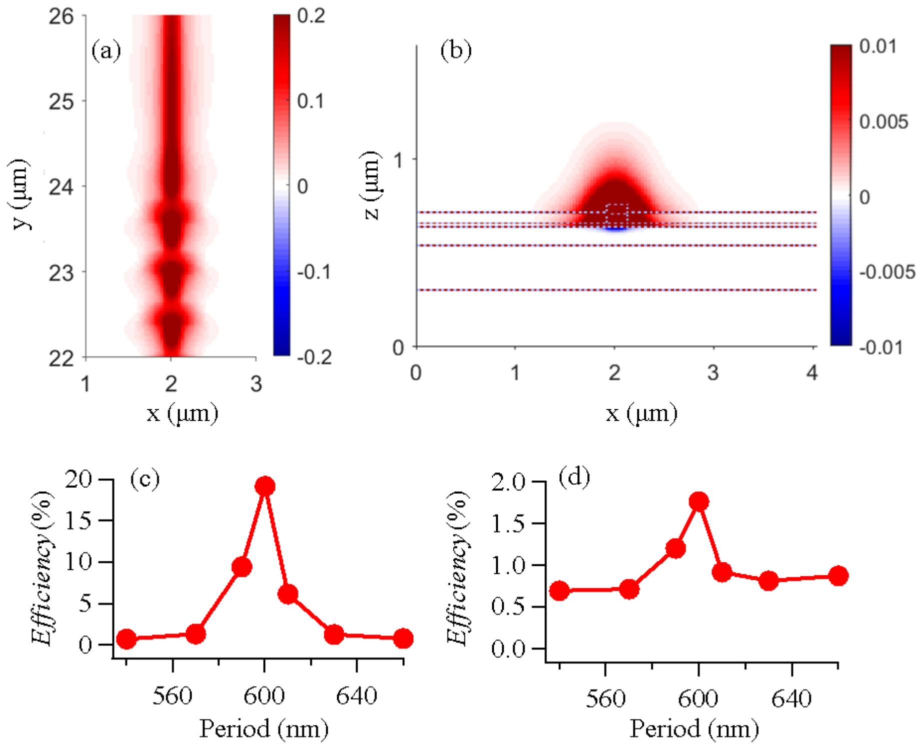

Fig. S14 The simulated efficiency of the SPP coupling. (a) The y-component S_y of the time-average Poynting vector $\vec{S}$ distribution at the SiO_2/Ag interface under the symmetric grating structure. (b) The cross-sectional view of the S_y-component of the time-average Poynting vector distribution of the symmetric grating structure at $y = 41\Lambda$. (c), and (d) The efficiency of the SPP coupling as a function of the grating period Λ with the symmetric grating and asymmetric grating structures, respectively.

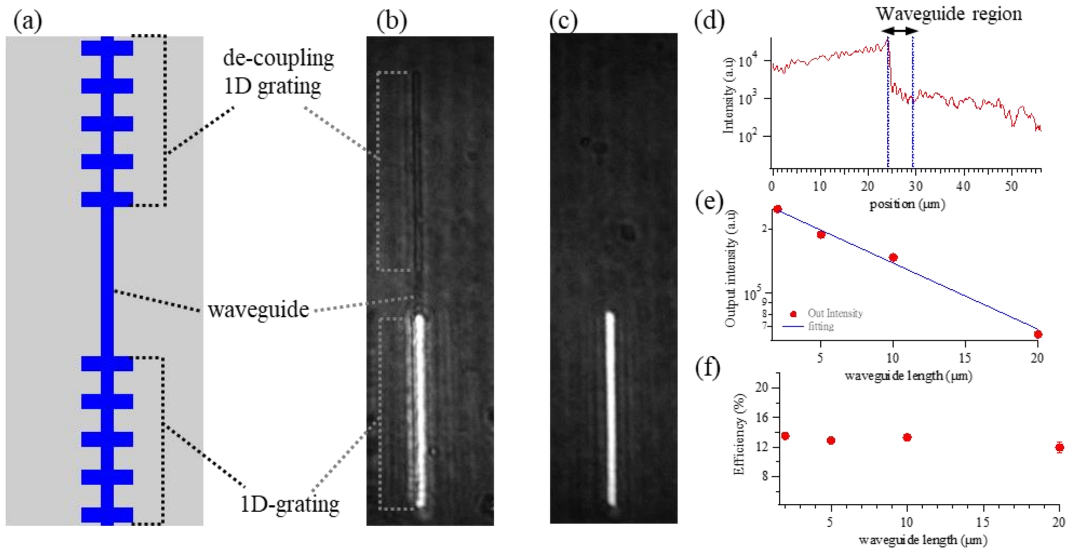

Figure S15 Characterization of SPP coupling efficiency using a dual- grating method. (a) The schematic of the sample design for SPP coupling efficiency measurement. The device design includes a 1-D grating for coupling 793-nm-wavelength light into the SPP, a TiO_2 waveguide of varying lengths for guiding SPP propagation toward the decoupling 1-D grating for converting the SPP wave back into light. The decoupling 1-D grating is identical to the 1-D coupling grating by design. (b) 793-nm-wavelength image of the sample with a 5-μm-long TiO_2waveguide. Only the coupling 1-D grating (brightest region) was illuminated with the incident light. The SPP decoupled into light is observed as a bright line at the decoupling 1-D grating region. (c) 793-nm-wavelength image of the sample with the incident light illuminating at blank area. (d) Line profile extracted from (b), with the TiO_2 waveguide region indicated by blue dashed lines. (e) Output intensity (the intensity collected over the decoupled grating) plotted as a function of the TiO_2waveguide length (red cicrles), fitted with an exponential decay (i.e. $y = y_0 + Ae^{-(x-x_0)/\tau}$; τ = 14.1 ± 1.3 μm). (f) Estimated coupling efficiency of the 1-D grating plotted as a function of the TiO_2 waveguide length.

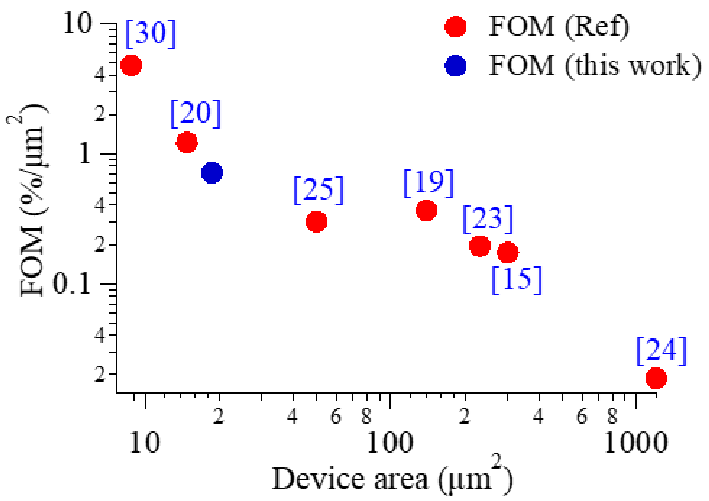

Fig. S16 FOM of SPP couplers plotted as function of their footprint area. The red filled circles represent those of the reported in the literature, and the blue filled circle that of this work. The bracketed numbering label indicate the reference number of the main text.

Ref.no	Area (μm^2)	SPP coupling efficiency (%)	FOM (%/μm^2)
[30]	8.8	42.0	4.7
[20]	14.8	18.0	1.2
[25]	50.0	15.0	0.30
[19]	140.0	51.0	0.36
[23]	230.4	45.0	0.20
[15]	300.0	52.0	0.17
[24]	1200.0	22.5	0.019
This work	18.7	13.4	0.71 ± 0.004

Table S1 FOM of the SPP couplers. The first column indicates the reference number of the main text.

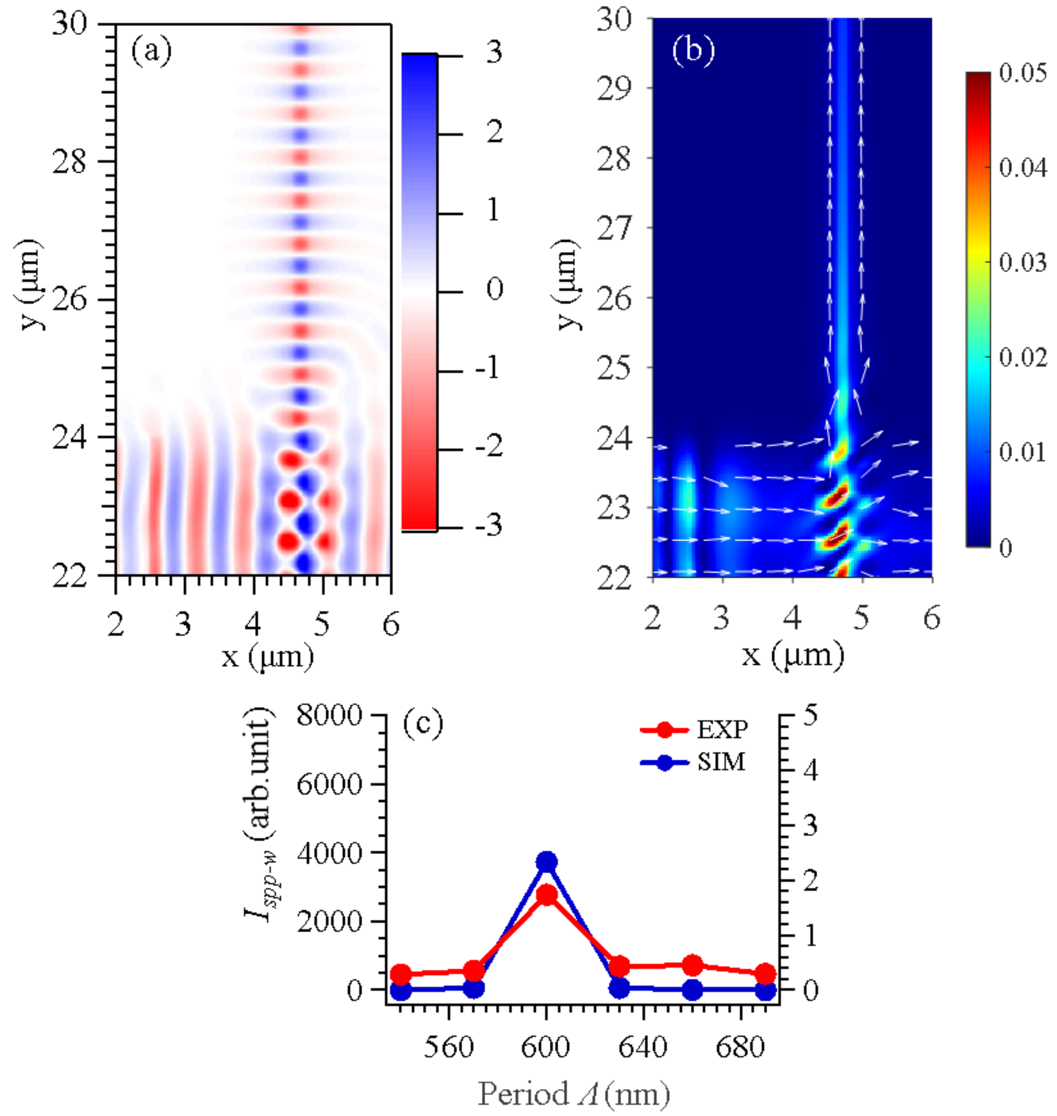

Fig. S17 The simulated SPP routing with the symmetric 1-D grating structure. (a) A snap shot of the simulated E_z-component of the E-field $|E|^2$ distribution at SiO_2/Ag interface. (b) The time-averaged Poynting vector distribution at the SiO_2/Ag interface. The arrows indicate the local direction of the Poynting vector. (c) The routed SPP intensity at the TiO_2 waveguide region as a function of the grating period $\boldsymbol{\Lambda}$.

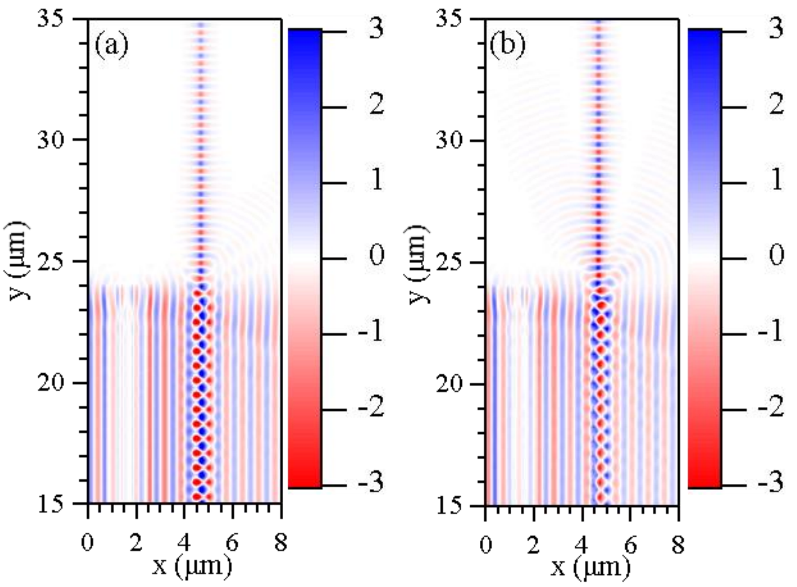

Fig. S18 The simulated SPP routing with the symmetric and asymmetric grating structure. (a) and (b), respectively. A larger field of view of the snap shot of the simulated E_z-component of the E-field $|E|^2$ distribution at SiO_2/Ag interface, including the grating coupler located between x = 0.5 μm and x = 2.5 μm. Due to limited computing resource, the number of the grooves in the linear grating couplers was decreased to 4 in the simulation, which is numerically sufficient to verify the coupling of the SPP at SiO_2/Ag interface.

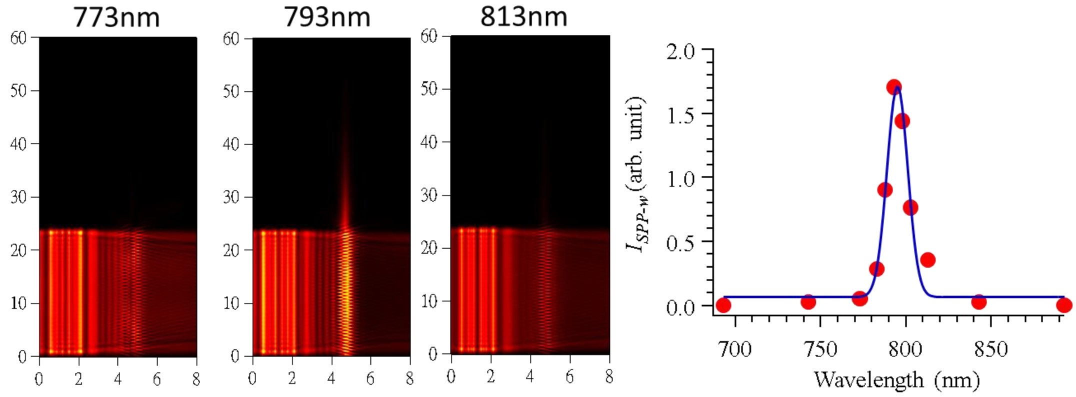

Fig. S19 The simulated incident light wavelength dependence of the SPP routing for the optimal asymmetric 1-D grating structure. (a) The E-field intensity $|E|^2$ for incident light as labelled. (b) the simulated (filled circle) SPP intensity at the TiO_2 waveguide as a function of the wavelength. The solid curve represents the result of a Gaussian fit to the data. The bandwidth according to the fitting parameter is ~14 nm.

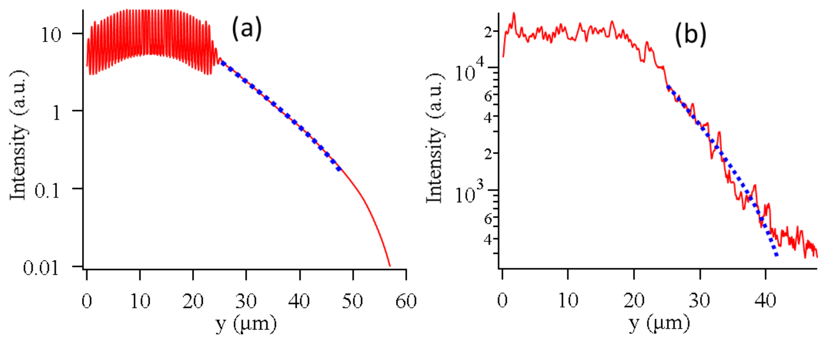

Fig. S20 Propagation distance dependence of the 90-degree routed SPP intensity along the 1-D TiO2 waveguide. (a) the $|E|^2$ intensity (solid curve) along the central TiO_2 stripe of the 1-D grating from the simulation shown in Fig. 6(b). The dashed line indicating an exponential fitting to the data, i.e. $I = I_0 + Ae^{-\frac{y-y_0}{\tau}}$ with $\tau = 8.37 \pm 0.02\ um$. (b) The up-conversion fluorescent intensity (solid curve) also extracted from the central TiO_2 stripe. The dashed line also represent the results of the same fitting with $7.3 \pm 0.3\ um$.

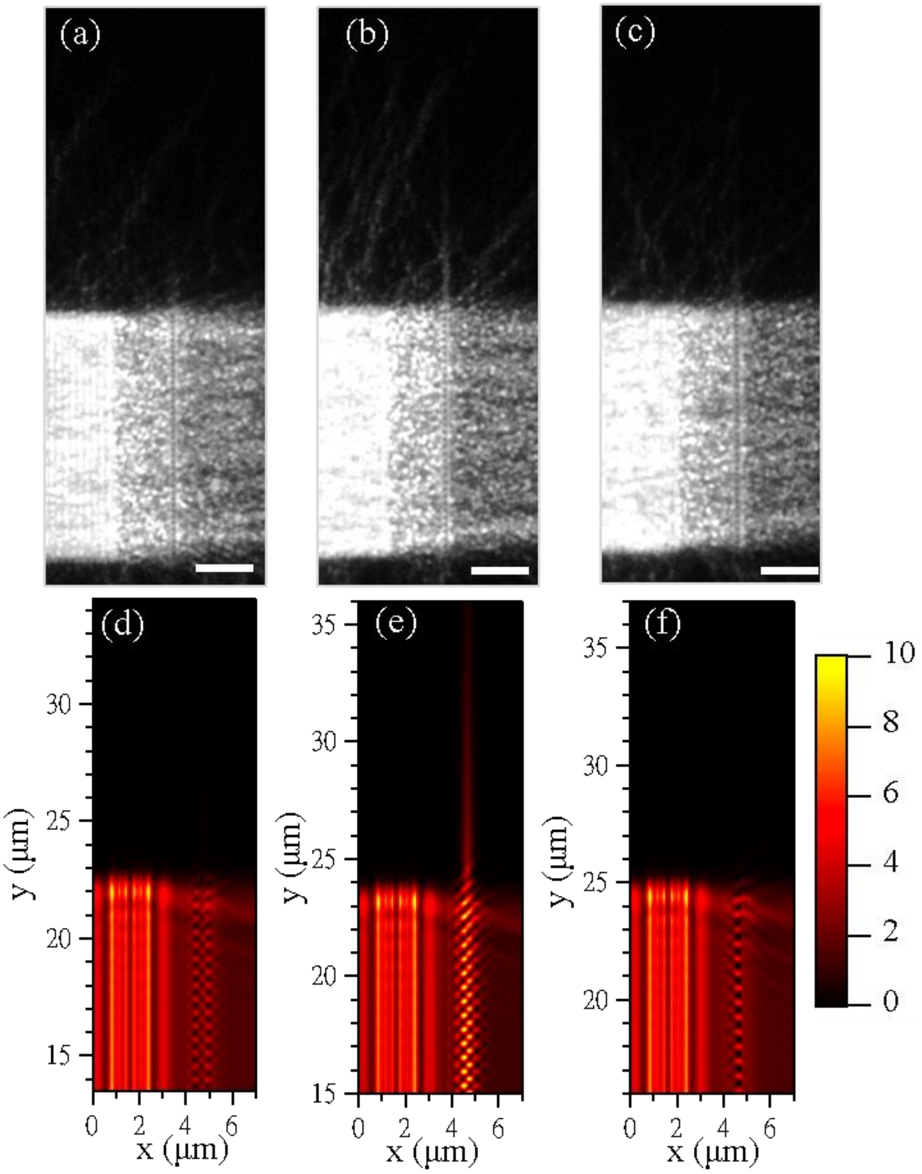

Fig. S21 (a), (b), and (c) The up-conversion fluorescent images of the SPP routing with the symmetric grating structures with the grating period of 570 nm, 600 nm, and 630 nm, respectively. (d), (e), and (f) The simulated *E*-field intensity $|E|^2$ distribution of the SPP routing with the symmetric grating structure with the grating period of 570 nm, 600 nm, and 630 nm, respectively. The size of the scale bar in (a)-(c) is 5 μm.

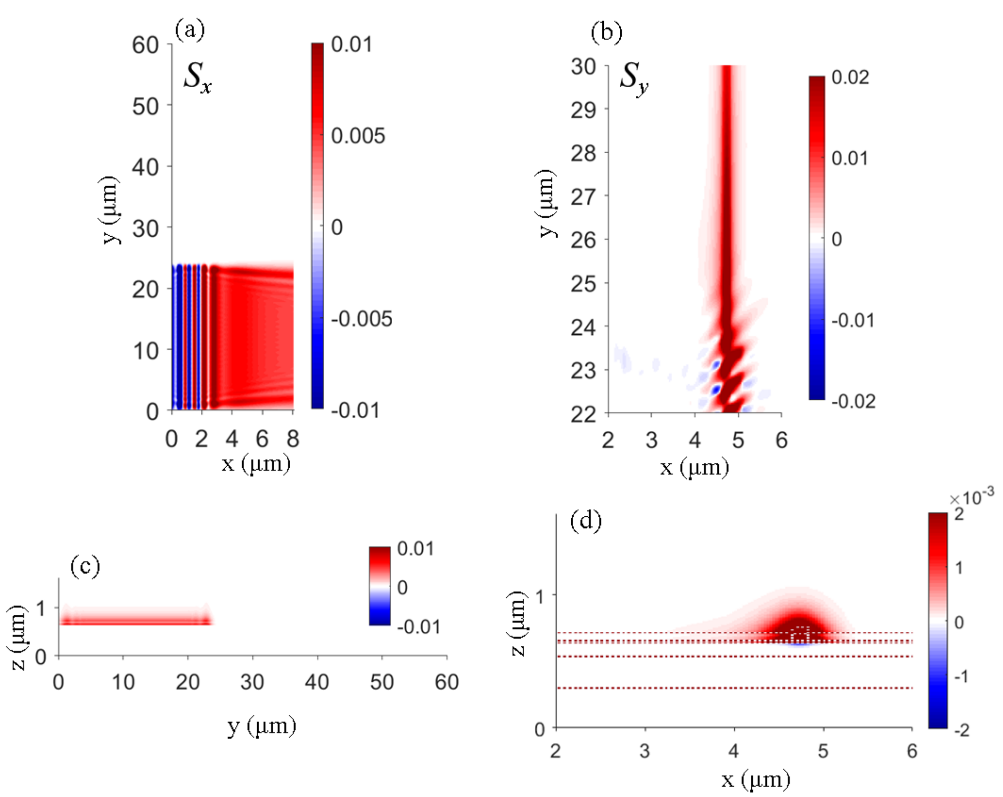

Fig. S22. The efficiency calculation in the FDTD simulation of the SPP routing. (a), (c) The S_x-component of the time-average Poynting vector for the incoming SPP without the 1-D grating structure in *x-y* plane and *y-z* plane, respectively. (b), (d) The S_y-component of the time-average Poynting vector for the SPP with the 1-D grating structure in *x-y* plane and *x-z* plane, respectively. The SPP incoming power is obtained by summing the values of the S_x-component in the *y-z* plane. The sum of the values of the S_y-component in the *x-z* plane is utilized as the output power for SPP routing. The efficiency is determined by dividing the SPP routing output power by the SPP incoming power in the simulation.

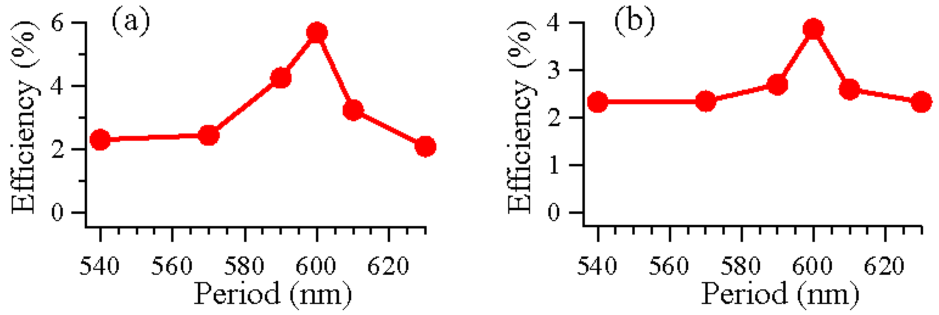

Fig. S23 (a), and (b) The efficiency of the SPP routing as a function of the grating period with the asymmetric and symmetric grating structure, respectively.

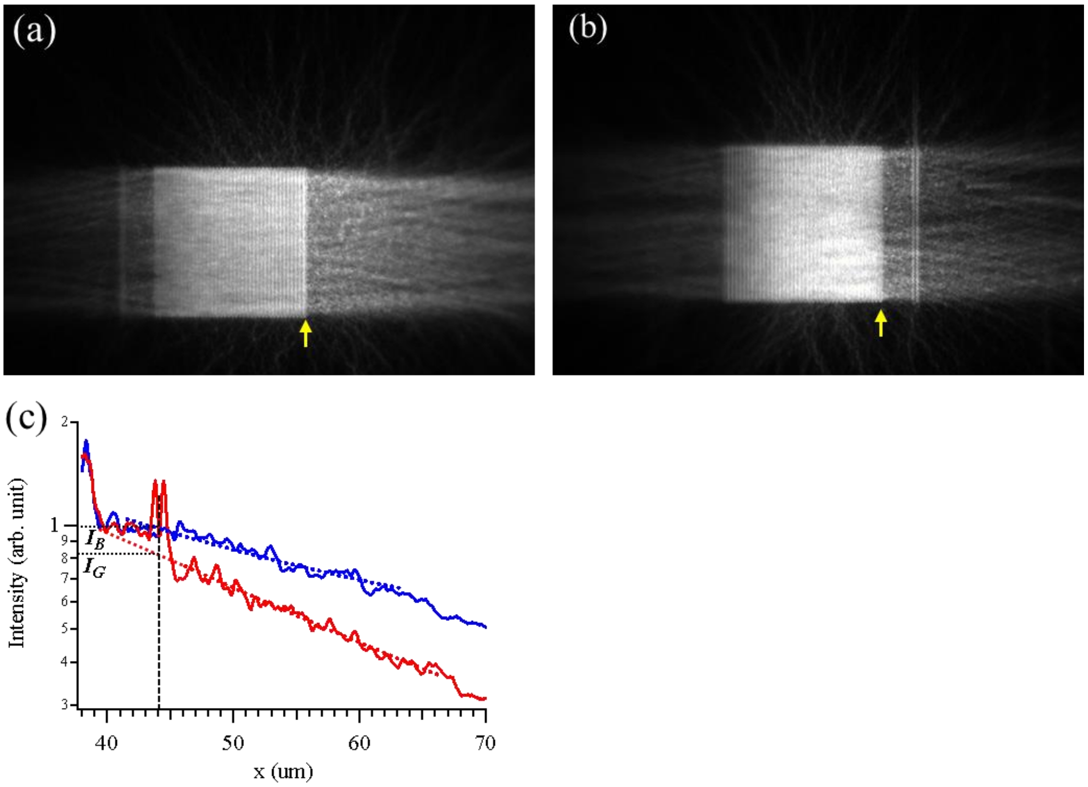

Figure S24. SPP router efficiency measurement. (a) Up-conversion fluorescent image of the 2-D SPP propagating at blank SiO_2/Ag interface. The linear grating (array of vertical bright lines) couples the incident to the 2-D SPP. (b) Up-conversion fluorescent image of the 2-D SPP passing the 1-D asymmetric grating connected with a 5-μm-long TiO_2 waveguide. (c) Horizontal intensity line profiles extracted from (a), the blue curve, and from (b), the red curve. The arrows in (a) and (b) mark the horizontal position of the starting point of the corresponding line profiles. The blue and red dashed lines represent the results of the exponential fitting to the respective line profiles.

Reference

[1] T. Holmgaard and S. I. Bozhevolnyi, "Theoretical analysis of dielectric-loaded surface plasmon polariton waveguides," Phys. Rev. B 75, 245405 (2007).
[2] L. Y. T. Nguyen, Y.-F. Chang, Y.-E. Tseng, H.-M. Chang, C.-C. Hsu, J.-Y. Lin and H.-C. Kan," Focusing of surface plasmon polaritons propagating at the SiO2/Ag interface with 2-level and 4-level Fresnel phase zone pad structures," Nanoscale, 15, 17198 (2023).
